# De novo transcriptome assembly of the cubomedusa *Tripedalia cystophora,* including the analysis of a set of genes involved in peptidergic neurotransmission

**DOI:** 10.1186/s12864-019-5514-7

**Published:** 2019-03-06

**Authors:** Sofie K. D. Nielsen, Thomas L. Koch, Frank Hauser, Anders Garm, Cornelis J. P. Grimmelikhuijzen

**Affiliations:** 1 Section of Marine Biology, Department of Biology, University of Copenhagen, Universitetsparken 4, 2100 Copenhagen, Denmark; 2 Section for Cell and Neurobiology, Department of Biology, University of Copenhagen, Universitetsparken 15, DK-2100 Copenhagen, Denmark

**Keywords:** Cnidaria, Cubozoa, Transcriptome, Vision, Opsin, Neuropeptide, Glycoprotein hormone, Biogenic amine, GPCR, LGR

## Abstract

**Background:**

The phyla Cnidaria, Placozoa, Ctenophora, and Porifera emerged before the split of proto- and deuterostome animals, about 600 million years ago. These early metazoans are interesting, because they can give us important information on the evolution of various tissues and organs, such as eyes and the nervous system. Generally, cnidarians have simple nervous systems, which use neuropeptides for their neurotransmission, but some cnidarian medusae belonging to the class Cubozoa (box jellyfishes) have advanced image-forming eyes, probably associated with a complex innervation. Here, we describe a new transcriptome database from the cubomedusa *Tripedalia cystophora.*

**Results:**

Based on the combined use of the Illumina and PacBio sequencing technologies, we produced a highly contiguous transcriptome database from *T. cystophora.* We then developed a software program to discover neuropeptide preprohormones in this database. This script enabled us to annotate seven novel *T. cystophora* neuropeptide preprohormone cDNAs: One coding for 19 copies of a peptide with the structure pQWLRGRFamide; one coding for six copies of a different RFamide peptide; one coding for six copies of pQPPGVWamide; one coding for eight different neuropeptide copies with the C-terminal LWamide sequence; one coding for thirteen copies of a peptide with the RPRAamide C-terminus; one coding for four copies of a peptide with the C-terminal GRYamide sequence; and one coding for seven copies of a cyclic peptide, of which the most frequent one has the sequence CTGQMCWFRamide. We could also identify orthologs of these seven preprohormones in the cubozoans *Alatina alata, Carybdea xaymacana, Chironex fleckeri,* and *Chiropsalmus quadrumanus.* Furthermore, using TBLASTN screening, we could annotate four bursicon-like glycoprotein hormone subunits, five opsins, and 52 other family-A G protein-coupled receptors (GPCRs), which also included two leucine-rich repeats containing G protein-coupled receptors (LGRs) in *T. cystophora*. The two LGRs are potential receptors for the glycoprotein hormones, while the other GPCRs are candidate receptors for the above-mentioned neuropeptides.

**Conclusions:**

By combining Illumina and PacBio sequencing technologies, we have produced a new high-quality de novo transcriptome assembly from *T. cystophora* that should be a valuable resource for identifying the neuronal components that are involved in vision and other behaviors in cubomedusae.

**Electronic supplementary material:**

The online version of this article (10.1186/s12864-019-5514-7) contains supplementary material, which is available to authorized users.

## Background

Cnidarians are basal, multicellular animals such as *Hydra*, corals, and jellyfishes. They are interesting from an evolutionary point of view, because they belong to a small group of phyla (together with Placozoa, Ctenophora, and Porifera) that evolved before the split of deuterostomes (e.g. vertebrates) and protostomes (most invertebrates, such as insects), an event that occurred about 600 million years ago [1]. Cnidarians have an anatomically simple nervous system, which consists of a diffuse nerve net that sometimes is condensed (centralized) in the head or foot regions of polyps, or fused as a giant axon in polyp tentacles, or as a giant nerve ring in the bell margins of medusae [2–13].

The nervous systems from cnidarians are highly peptidergic: A large number of cnidarian neuropeptides have been chemically isolated and sequenced from cnidarians and their preprohormones have been cloned [14–33].

The cnidarian preprohormones often contain a high number of immature neuropeptide copies, ranging from 4 to 37 copies per preprohormone molecule [16–18, 20, 21, 23, 26, 27, 29, 33]. Each immature neuropeptide copy is flanked by processing signals: At the C-terminal sides of the immature neuropeptide sequences, these signals consist of the amino acid sequences GKR, GKK, or GR(R). The Arg (R) and Lys (K) residues are recognized by classical prohormone convertases (PC-1/3 or PC-2), which liberate the neuropeptide sequences, while the Gly (G) residues are converted into C-terminal amide groups by the enzyme peptidylglycine α-amidating monooxygenase [29, 34–36].

At the N-terminal sides of the immature cnidarian neuropeptide sequences, we very often find a Gln (Q) residue, which is cyclized into a pyroglutamate group (pQ) and which protects the N-terminus of the neuropeptide against enzymatic degradation [16–18, 20, 21, 29]. In contrast to higher metazoans, however, the N-terminal processing sites preceding these Q residues are normally not dibasic residues, but often acidic (E or D) residues, or T, S, N, L, or V residues, suggesting the existence of novel endo- or aminopeptidases carrying out processing of cnidarian preprohormones [16–18, 20, 29]. These findings make it sometimes difficult to predict the N-terminus of a mature neuropeptide sequence from a cloned neuropeptide preprohormone. If a Q residue is found N-terminally of a PC 1/3 cleavage site preceded by acidic (E, D) or T, S, N, L or V residues, cleavage probably occurs N-terminally of this Q residue, yielding a protecting N-terminal pyroglutamate residue.

Cnidarian neuropeptides have a broad spectrum of biological activities, including stimulation of the maturation and release of oocytes (spawning) in hydrozoan medusa, stimulation or inhibition of metamorphosis in hydrozoan planula larvae, stimulation of nerve cell differentiation in hydrozoan polyps, and stimulation or inhibition of smooth muscle contractions in hydrozoans and sea anemones [28, 32, 33, 37–46].

In proto- and deuterostomes, neuropeptides normally act on G protein-coupled receptors (GPCRs), which are transmembrane proteins located in the cell membrane [47]. In cnidarians, one such GPCR has recently been identified (deorphanized) as the receptor for a hydromedusan neuropeptide that stimulates oocyte maturation [33]. GPCRs are metabotropic receptors that transmit their activation via second messengers and, because of the many steps involved, act relatively slowly. In cnidarians, however, some neuropeptides activate ionotropic receptors, such as the hydrozoan RFamide neuropeptides, which activate trimeric cell membrane channels belonging to the degenerin/epithelial Na^+^ channel (DEG/ENaC) family [48–52]. This peptidergic signal transmission via ligand-gated ion channels can be very fast.

Cnidarians probably also use protein hormones for their intercellular signaling. Already 25 years ago, we were able to clone a protein hormone receptor from sea anemones that was structurally closely related to mammalian glycoprotein receptors such as the ones that are activated by follicle stimulating hormone (FSH), luteinizing hormone (LH), or thyroid stimulating hormone (TSH) [53, 54]. Glycoprotein hormones are normally heterodimers. Such dimer subunits, however, have not been identified from cnidarians, so far.

Finally, cnidarians also use biogenic amines as neurotransmitters [55] and we have recently identified (deorphanized) a GPCR from *Hydra magnipapilla* that was a functional muscarinic acetylcholine receptor [56, 57]. The occurrence of this receptor gene, however, appears to be confined to hydrozoans and does not exist in other cnidarians [57].

The phylum Cnidaria is generally subdivided into six classes: Hydrozoa (*Hydra* and colonial hydrozoans, such as *Hydractinia*), Anthozoa (such as sea anemones and corals), Scyphozoa (jellyfishes), Staurozoa (stalked jellyfishes), Cubomedusa (box jellyfishes), and Myxozoa (small obligate parasites). The nervous systems in animals belonging to these six classes all have the above-mentioned properties, for example they are all peptidergic, and their anatomy is diffuse with occasional centralizations [3–11]. However, many cubozoans, such as *Tripedalia cystophora,* have complex eyes, grouped together as six eyes on each of the four rhopalia, of which two eyes (the upper and lower lens eyes) are camera-type, image-forming eyes. These lower lense eyes are even able to adjust their pupils to light intensity [58–61]. One can expect that the innervation of these eyes and their signal processing must be unusually complex compared to the more basal signal transmission, occurring in other non-cubozoan cnidarians.

In our current paper, we are presenting a highly contiguous transcriptome database from *T. cystophora,* which was based on the combined use of Illumina and PacBio sequencing, that could help us to identify the neuronal components that are involved in the innervation and processing of vision in cubomedusae. We have also compared the quality of our transcriptome with that of other cubozoan transcriptomes, which showed that our transcriptome was of high quality. Finally, we have tested the transcriptome and identified a set of novel genes involved in peptidergic neurotransmission.

## Results

### De novo transcriptome by PacBio sequencing

We isolated RNA from 12 *T. cystophora* medusae, converted it into cDNA, and sequenced it, using the PacBio (Pacific Biosciences) sequencing technology (Additional file 1A-D). Comparison of this PacBio database with the Illumina reads (see below) gave us the information that some transcripts were missing in the PacBio database. We, therefore, carried out a second PacBio sequencing round of the same *T. cystophora* cDNA sample as mentioned above with the expectation that this would improve the completeness of the combined PacBio data set (Additional file 2A-C). All parameters in this second sequencing round were the same as in the first round. This second sequencing round improved our dataset considerably. In the following we give the combined data from the first and second sequencing rounds: Reads of interest (ROI; for definition see Additional file 1A), 645,865; containing 275,377 (42.64%) full length non-chimeric transcripts. After the Quiver polishing procedure (see Methods) we ended up with 88,588 high quality transcripts (mean quality index > 0.99) and 106,394 low quality transcripts (mean quality index of 0.30). For length distribution of ROI’s and the definition of quality index, see Additional files 1A and 2A. The coverage of the high quality pool was 44 reads/transcript, while the coverage of the low quality pool was 9 reads/transcript (for further details, see Additional files 2A-C). We ended up with 46,348 unique transcripts (also called unigenes) after redundancy removal. A PacBio pipeline output summary is given in Additional file 2C.

### Error correction of the PacBio transcripts using Illumina reads

We also sequenced around 223 million paired-end reads from the Illumina X Ten platform, using *T. cystophora* cDNA derived for the same sample as the PacBio data. Around 204 million clean reads were generated, of which 99.3% had a base accuracy of 99 and 97.7% reads had a base accuracy of 99.9%. For an RNA-Seq pipeline outcome summary and quality assessment see Additional file 3. These short reads were subsequently used for correcting the PacBio consensus isoform sequences following two error correction pipelines, Proovread and LoRDEC (long read de Bruijn graph error correction) [62, 63] (see Additional file 4A and B).

### Comparison of the *T. cystophora* transcripts with a set of eukaryotic universally conserved orthologues

In Additional file 5A-E we have compared the assembled transcripts of our *T. cystophora* transcriptome with those from other eukaryotes. From a Venn diagram (Additional file 5E), which can be regarded as an estimate of transcript assembly quality, one can conclude that from the 46,348 unigenes (transcripts) present in our database, 23,286 unigenes had universally conserved ortholog genes in common with the SwissProt, InterPro, Kyoto Encyclopedia of Genes and Genomes, and Eukaryotic Orthologue Group databases (=50%). These numbers compare well with other transcriptome databases.

### Annotations of transcripts coding for neuropeptide preprohormones

Most cnidarian neuropeptide preprohormones have basic cleavage sites (KR, RR) at the C-terminal parts of their immature neuropeptide sequences, preceded by a glycine (G) residue, which, after cleavage of the preprohormone, is converted into a C-terminal amide group [21, 29]. Furthermore, cnidarian preprohormones very often have multiple copies of the immature neuropeptide sequences [21, 29]. Therefore, we wrote a software program in Python3 that was based on these preprohormone features and that only filtered protein-coding sequences from the transcriptome database that contained at least three similar amino acid sequences, each ending with the sequence GKR, GKK, or GR. The flow chart of our program is given in Additional file 6 and the software is given in Additional file 7. Furthermore, we have deposited our software at [64].

**Fig. 1:**
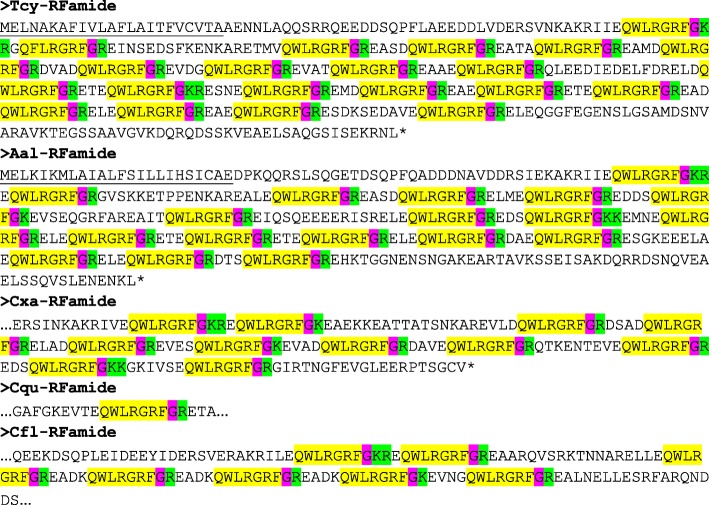
Amino acid sequences of the RFamide preprohormone from *T. cystophora* (Tcy-RFamide), *A. alata* (Aal-RFamide), *C. xaymacana* (Cxa-RFamide), *C. quadrumanus* (Cqu-RFamide), and *C. fleckeri* (Cfl-RFamide). In the complete proteins, the signal peptides are underlined and the stop codons are indicated by asterisks. Prohormone convertase (PC 1/3) cleavage sites (KR, R, KK) are highlighted in green and the C-terminal G residues, which are converted into C-terminal amide groups by peptidyl-glycine α-amidating monooxygenase, are highlighted in red. The above-mentioned processing enzymes liberate peptide fragments (highlighted in yellow) with the C-terminal sequence RFamide. The N-termini of these peptides are determined by Q residues that we assume are converted into protective pyroglutamate residues (pQ) by the enzyme glutaminyl cyclase. These Q residues are often preceded by acidic residues (D or E), which are established processing sites in cnidarians, but not in higher metazoans [21, 29]. These actions would yield 19 copies of Tcy-RFamide-1 (pQWLRGRFamide), and one copy of Tcy-RFamide-2 (pQFLRGRFamide), which are N-terminally protected by pQ residues and C-terminally by amide groups (see also Table 1). In the Aal-RFamide preprohormone (second panel from the top) there are 18 copies of a peptide identical to Tcy-RFamide-1 (see also Table 1). These peptide sequences are preceded nearly exclusively by acidic (D and E) and occasionally by S residues. In the incomplete Cxa-RFamide preprohormone 11 copies of a peptide identical to Tcy-RFamide-1 are present (see also Table 1). Most peptide sequences are preceded by acidic residues, while two peptide sequences are preceded by S residues. From *C. quadrumanus* (fourth panel from the top) we could only identify a short incomplete preprohormone fragment, containing one copy of a peptide sequence identical to Tcy-RFamide-1. This copy is preceded by an acidic (E) residue. Finally, the incomplete *C. fleckeri* preprohormone (bottom panel) contains seven copies of a peptide identical to Tcy-RFamide-1. Most copies are preceded by acidic residues, while one copy is preceded by a G and other copies by K residues

**Fig. 2:**
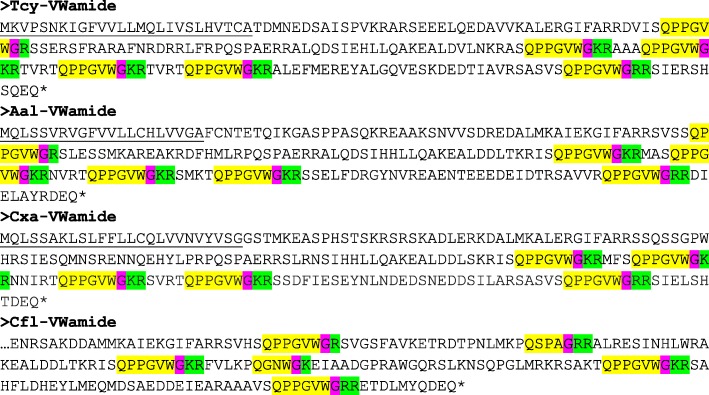
Amino acid sequences of the complete VWamide preprohormone from *T. cystophora, A. alata, C. xaymacana,* and *C. fleckeri*. Residues and peptide sequences are highlighted as in Fig. 1. The VWamide preprohormone from *T. cystophora* (named Tcy-VWamide) contains six copies of Tcy-VWamide-1 (pQPPGVWamide), which are preceded by wither S, T, or A residues. The VWamide preprohormone from *A. alata* contains six copies of a neuropeptide identical to Tcy-VWamide-1, which are preceded by either S, T, or R residues. The VWamide preprohormone from *C. xaymacana* contains five copies of Tcy-VWamide-1. Each copy is preceded by either S, or T residues. The VWamide preprohormone from *C. fleckeri* contains four copies of Tcy-VWamide-1, one copy of a peptide with the PAamide C-terminal sequence (pQSPAamide), and one copy of a peptide with the NWamide C-terminal sequence (pQGNWamide)

**Fig. 3:**
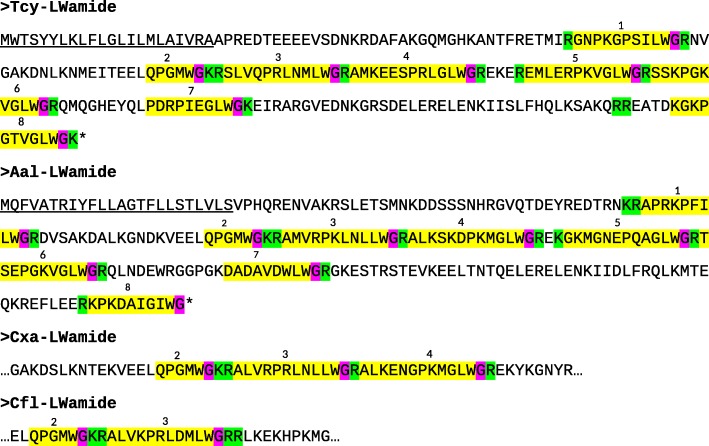
Complete or partial acid sequences of four LWamide preprohormones from *T. cystophora, A. alata, C. xaymacana,* and *C. fleckeri.* Residues and peptide sequences are highlighted as in Fig. 1. These preprohormones can be processed into a number of peptides with either the LWamide or MWamide C-terminus, while the N-termini of some of these peptides are somewhat uncertain (Table 1). Interestingly, the second neuropeptide sequence (counted from the N-terminus), pQPGMWamide, is completely identical in all four cubomedusan preprohormones. These sequences are preceded by L residues, which again would imply processing C-terminally from L [21, 29]. Similarly, the third peptide sequence (counted from the N-terminus) from each preprohormone constitute a peptide subfamily of nearly identical sequences. Table 2 gives our proposal for their structures, although there are uncertainties about their N-termini. The proposed peptide subfamilies have discrete structures, which enable us to identify the first peptide sequences in the *C. xaymacana* and *C. fleckeri* preprohormone fragments as peptides-2 (belonging to peptide family-2) followed by peptides-3 and -4

**Fig. 4:**
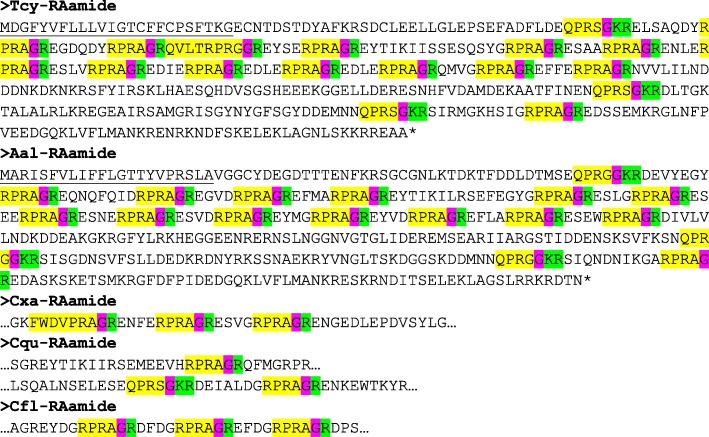
Amino acid sequences of the RAamide preprohormones from *T. cystophora, A. alata, C. xaymacana, C. quadrumanus,* and *C. fleckeri.* Residues and peptide sequences are highlighted as in Fig. 1. In *T. cystophora*, the preprohormone (named Tcy-RAamide) can be processed into 13 peptide copies with the C-terminal sequence RPRAamide, (named Tcy-RAamide-1), three copies with the sequence pQPRSamide (named Tcy-RSamide-1), and one copy with the sequence pQVLTRPRGamide (named Tcy-RGamide; see Table 1). In *A. alata,* the preprohormone (named Aal-RAamide) contains 14 peptide copies with the RPRAamide C-terminus, and three copies of pQPRGamide (Table 1). In *C. xaymacana*, we identified a small preprohormone fragment (named Cxa-RAamide) that contained two peptide copies with the RPRAamide C-terminal sequence, and one copy with the VPRAamide C-terminal sequence (Table 1). In the *C. quadrumanus* transcriptome we identified two small preprohormone fragments (probably part of one preprohormone named Cqu-RAamide) that contained two copies of a RPRAamide peptide and one peptide with the sequence pQPRSamide (Table 1). In *C. fleckeri* we identified a small preprohormone fragment (named Cfl-RAamide) that contained three copies of a peptide with the C-terminal sequence RPRAamide (Table 1)

**Fig. 5:**
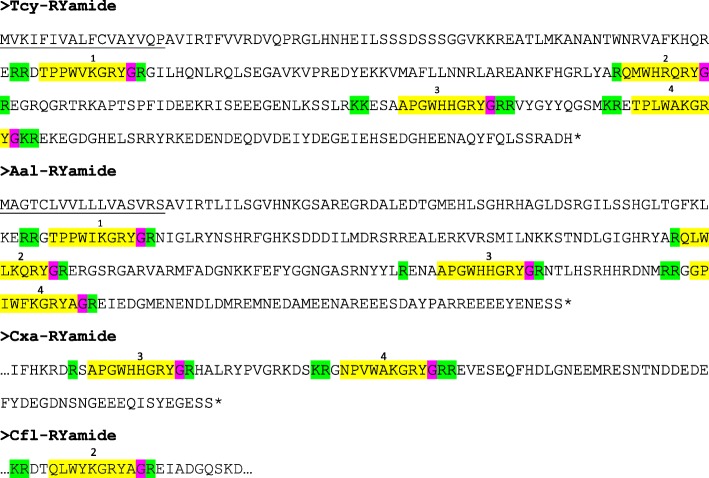
Amino acid sequences of the RYamide preprohormones from *T. cystophora, A. alata, C. xayamacana,* and *C. fleckeri.* Residues and peptide sequences are highlighted as in Fig. 1. These preprohormones can be processed in a number of neuropeptides with the C-terminal RYamide sequences. Just like the LWamide preprohormones (Fig. 3), it is possible to group these peptides into four peptide subfamilies which, interestingly, are positioned at discrete locations on the medusa RYamide preprohormones (Table 3). At positions-1 (counted from the N- to the C-terminus) the the *T. cystophora* and *A. alatina* preprohormones contain two nearly identical RYamide neuropeptides (TPPWVKGRYamide, respectively TPPWIKGRYamide; see also Table 3). At positions-2 (counted from the N- to the C-terminus) the *T. cystophora*, *A. alatina,* and *C. fleckeri* preprohormones contained three highly similar neuropeptides, which in *T. cystophora* has the sequence pQMWHRQRYamide (see also Table 3). At positions-3 (counted from the N- to C-terminus), the preprohormones from *T. cystophora, A. alata,* and *C. xaymacana* each contains an identical neuropeptide with the sequence APGWHHGRYamide (see also Table 3). At positions-4, the preprohormones from *T. cystophora, A. alata,* and *C. xaymacana* contain sequences of closely related peptides of which the one in *T. cystophora* has the sequence TPLWAKGRYamide (see also Table 3). The four neuropeptide subfamilies have structural signatures (Table 3), which enable us to assign a peptide-3 (belonging to the third peptide subfamily) and a peptide-4 (belonging to the fourth peptide subfamily) on the preprohormone fragment from *C. xaymacana* and a peptide-2 (belonging to the second peptide subfamily) on the small preprohormone fragment from *C. fleckeri*

**Fig. 6:**
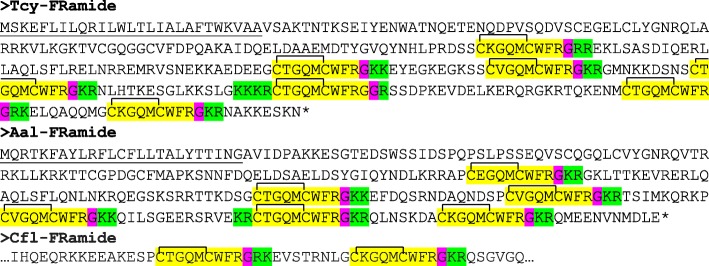
Amino acid sequences of the FRamide preprohormones from *T. cystophora, A. alata,* and *C. fleckeri.* Residues and peptide sequences are highlighted as in Fig. 1. The *T. cystophora* preprohormone produces four copies of a neuropeptide with the sequence CTGQMCWFRamide (named Tcy-FRamide-1), two copies of CKGQMCWFRamide (Tcy-FRamide-2), and one copy of CVGQMCWFRamide (Tcy-FRamide-3). The preprohormone from *A. alata* produces two copies of a peptide identical to Tcy-FRamide-1, two copies of a peptide identical to Tcy-FRamide-3, one copy of a peptide identical to Tcy-FRamide-2, and one copy of CEGQMCWFRamide (Table 1). The preprohormone fragment from *C. fleckeri* contains one copy of a peptide identical to Tcy-FRamide-1 and one peptide copy identical to Tcy-FRamide-2 (Table 1). All peptides contained in the three preprohormones are nearly identical in structure and only vary in the second amino acid residue, being either T, K, V, or E

The application of our software program to the combined *T. cystophora* transcriptome databases (PacBio first and second round, and Illumina databases) detected seven putative neuropeptide preprohormones. Furthermore, many of these preprohormones could also be detected in transcriptomes from other cubozoan species:

(i) One complete preprohormone (having both a signal sequence and a stop codon in its cDNA) containing 19 copies of the neuropeptide sequence pQWLRGRFamide (named Tcy-RFamide-1) and one copy of pQFLRGRFamide (named Tcy-RFamide-2) is present in the database from *T. cystophora* (Fig. 1, Table 1). It is interesting that, like in other cnidarian RFamide preprohormones [21, 29], these neuropeptide sequences are very often preceded by acidic (D or E) residues, suggesting that these residues are processing sites and that the proposed neuropeptide sequences are correct.
  Similarly, we found a complete RFamide preprohormone in the transcriptome database from *A. alata* [65] that contained 18 copies of the neuropeptide pQWLRGRFamide, which is identical to Tcy-RFamide-1 (Fig. 1, Table 1). Also here, most neuropeptide sequences are preceded by acidic (D, E) residues, while two sequences are preceded by S residues (Fig. 1).
  In the transcriptome database from the cubomedusa *Carybdea xaymacana*, we could identify an incomplete RFamide preprohormone (lacking the signal sequence) that contained 11 copies of a neuropeptide sequence that was identical to Tcy-RFamide-1 (Fig. 1, Table 1). This incompleteness of the preprohormone was likely due to multiple gaps present in the *C. xaymacana* Illumina transcriptome.
  Similarly, the transcriptome assembly from the cubomedusa *Chiropsalmus quadrumanus* contained an incomplete preprohormone, having one copy of a neuropeptide identical to Tcy-RFamide-1 (Fig. 1, Table 1).
  Finally, the transcriptome database from the cubomedusa *Chironex fleckeri* contained one incomplete preprohormone sequence coding for seven RFamide neuropeptides that were identical to Tcy-RFamide-1 (Fig. 1, Table 1). Three of these neuropeptide sequences were preceded by acidic residues, while three of them were preceded by K and one by G (Fig. 1).
(ii) We discovered a second potential RFamide preprohormone in our *T. cystophora* database named Tcy-RFamide-II (Additional file 8, Table 1). This preprohormone is complete, including a signal peptide, but we are unsure about the final mature structures of the biologically active peptides. Because PC 1/3-mediated processing could occur in between the RRR sequences (Additional file 8), the most likely products are six copies of RFamide. These RFamide sequences are very short compared to other known neuropeptides. For example, the shortest mammalian neuropeptide known is the tripeptide thyrotropin-releasing-hormone (TRH), pQHPamide [66], which, in contrast to the RFamide peptide, is N-terminally protected. We are, therefore, skeptical about the preprohormone status of Tcy-RFamide-II.
  A similar preprohormone as Tcy-RFamide-II can be identified in the *A. alata* database. Because this database only consists of Illumina reads, the complete preprohormone was difficult to assemble and the protein remained, therefore, incomplete (Additional file 8, Table 1).
  No RFamide-II preprohormones could be identified in the transcriptome databases from the other cubomedusae.
(iii) In our *T. cystophora* transcriptome we could annotate a complete preprohormone that contained six copies of the proposed neuropeptide pQPPGVWamide (named Tcy-VWamide-1; Fig. 2, Table 1). Five of these neuropeptide sequences are preceded by either S or T residues, a phenomenon that we observed earlier [21, 29] suggesting, again, processing at unusual amino acid residues.
  A preprohormone that contained six copies of a neuropeptide that was identical to Tcy-VWamide-1 could also be annotated from the transcriptome of *A. alatina* (Fig. 4, Table 1). Also here, most neuropeptide sequences are preceded by either S or T residues, suggesting unusual processing.
  Also, in the transcriptome of *C. xaymacana* we could identify a complete preprohormone that contained five copies of a neuropeptide identical to Tcy-VWamide-1 (Fig. 2, Table 1).
  In addition, we could identify an incomplete preprohormone in the transcriptome from *C. fleckeri* that contained four neuropeptide copies identical to Tcy-VWamide-1. This precursor might also contain two other neuropeptide sequences that are different from Tcy-VWamide-1 (Fig. 2, Table 1).
  We could not find a VWamide preprohormone in the transcriptome of *C. quadrumanus*, probably due to insufficient sequencing depth.
(iv) We could annotate a complete preprohormone in *T. cystophora* (named Tcy-LWamide) that contained seven neuropeptide copies with the C-terminal amino acid sequence LWamide and one copy of a peptide with the C-terminal MWamide sequence (Fig. 3, Table 1). For this preprohormone, it is difficult to predict the N-termini of each neuropeptide sequence, due to the uncertainties of N-terminal neuropeptide processing (Fig. 3, Table 1; see, however, below).
  A similar complete preprohormone can be predicted from the transcriptome of *A. alata* (Fig. 3, Table 1), which has six copies of an LWamide, one copy of a MWamide, and one copy of an IWamide neuropeptide.
  The transcriptomes from *C. xaymacana,* and *C. fleckeri* only contain incomplete fragments of an LWamide preprohormone, having one to three copies of the LWamide or MWamide neuropeptides (Fig. 3, Table 1).
  When we aligned the LWamide preprohormones from the four cubomedusa species, we could see that they contained descrete LWamide or MWamide peptide subfamilies that were lying in a certain order from the N- to the C-termini. For example, peptide-2 (the second peptide from the N-terminus) in the preprohormones from *T. cystophora, A. alata, C. xaymacana,* and *C. fleckeri* always had the sequence ELQPGMWamide. When we would accept the existence of a hypothetical aminopeptidase processing C-terminally from the L residue [21], this subfamily would consist of four identical copies of pQPGMWamide (Table 2). Thus, each cubomedusan species would contain one copy of this predicted peptide situated at peptide position-2 of the LWamide preprohormone. Peptide-3 (the third peptide from the N-terminus) always had the sequence A(or S)L(or M)VR(or K, or Q)PR(or K)LNL(or M)LWamide. This, then, is again a discrete peptide subfamily with a PRL or PKL core and an LWamide C-terminus (Table 2). Peptides-4 and -5, however (the fourth and fifth peptide from the N-terminus) have the C-terminus PR(or K)L(or M, V, or A)GLWamide and appear, therefore, to be related to each other (Table 2). Peptide-6 (the sixth peptide from the N-terminus in the preprohormone) always has the C-terminal sequence PGKVGLWamide, which is different from the peptides located at the other positions (Table 2). In conclusion, discrete sequence signatures can be recognized in the peptide subfamilies positioned at peptide positions 1, 2, 3, 4/5, and 6 (Table 2). We call the peptides belonging to these subfamilies peptide-1 to − 6 and not LWamide-1 to − 6, because the peptides belonging to family-2 have the C-terminus MWamide.
  Because of these discrete sequence signatures, the preprohormone fragments from *C. xaymacana* and *C. fleckeri* (Fig. 3) can easily be identified as a fragment containing (counted from the N- to the C-terminus) one copy of a peptide-2, − 3, and − 4 (*C. xaymacana*); and a fragment containing one copy of a peptide-2 and -3 sequence (*C. fleckeri*).
(v) In the transcriptome from *T. cystophora* we could annotate a complete preprohormone (name: Tcy-RAamide; Fig. 4) that contained thirteen copies of an RPRAamide neuropeptide, three copies of a PRSamide, and one copy of a PRGamide neuropeptide. In two cases (pQPRSamide, the first peptide, and pQVLTRPRGamide, the fourth peptide sequence counted from the N-terminus of the preprohormone, Fig. 4), the mature structures of the neuropeptide sequences can be readily predicted, because their Q residues are preceded by an acidic (E) or basic (R) residue, while for the other neuropeptide sequences the N-termini are uncertain (Fig. 4, Table 1).
  A similar complete RAamide preprohormone can be identified in the transcriptome from *A. alatina* (named Aal-RAamide, Fig. 4, Table 1). This preprohormone contains fourteen copies of a neuropeptide with the C-terminal sequence RPRAamide and three copies of a neuropeptide with a QPRGamide C-terminus. These last three peptides might have the mature structure pQPRGamide, while the N-termini of the other peptides are uncertain (Fig. 4).
  In the transcriptome from *C. xaymacana, C. quadrumanus,* and *C. fleckeri*, we identified incomplete RAamide preprohormone fragments that contained between one and three copies of an RAamide or RSamide neuropeptide (Fig. 4, Table 1). One of the peptides located on the second preprohormone fragment from *C. quadrumanus* (Fig. 4) is preceded by an acidic residue and has the likely structure pQPRSamide (Table 1).
(vi) We identified a complete RYamide preprohormone (named Tcy-RYamide) in the transcriptome from *T. cystophora* that contained four copies of an RYamide neuropeptide.
  The first peptide located near the N-terminus of the preprohormone (Fig. 5) (named Tcy-RYamide-1) has the likely sequence TPPWVKGRYamide and is protected at its N-terminus by the two proline residues at positions 2 and 3 (protective imide bonds between residues 1 and 2, and 2 and 3) (Tables 1 and 3). The second peptide counted from the N-terminus of the preprohormone has the sequence pQMWHRQRYamide (named Tcy-RYamide-2) and is protected at its N-terminus by a pyroglutamate residue (Fig. 5, Tables 1 and 3). The third peptide counted from the N-terminus has the likely sequence APGWHHGRYamide (named Tcy-RYamide-3) and is N-terminally protected by its proline residue at amino acid position-2 (Fig. 5, Tables 1 and 3). The fourth peptide counted from the N-terminus has the probable sequence TPLWAKGRYamide and is protected at its N-terminus by the proline residue at amino acid position-2 (Fig. 5, Table 1 and 3).
  In the transcriptome from *A. alatina* we could also annotate a complete preprohormone that was very similar to the RYamide preprohormone from *T. cystophora* (Fig. 5). When counted from the N- to the C-terminus of this preprohormone, we could identify a peptide-1 that was nearly identical to Tcy-RYamide-1; a peptide-2 that was nearly identical to Tcy-RYamide-2; a peptide-3 that was completely identical to Tcy-RYamide-3: and a peptide-4 that was nearly identical to Tcy-RYamide-4 (Fig. 5, Tables 1 and 3).
  In the transcriptome from *C. xaymacana* we could annotate an RY-amide preprohormone fragment that contained one copy of a peptide that was identical to Tcy-RYamide-3, and one that was very similar to Tcy-RYamide-4 (Fig. 5, Table 3).
  In the *C. fleckeri* transcriptome we could annotate an RYamide preprohormone fragment that contained one copy of a peptide that was very similar to Tcy-RYamide-2 (Fig. 5, Table 3).
(vii) We could annotate a complete preprohormone in the Illumina Sequence Read Archive, SRA (NCBI accession number SRR779134), but not in the PacBio or Transcriptome Shotgun Assembly (TSA) database of *T. cystophora* that contains seven similar copies of an FRamide peptide (Fig. 6, Table 1). Four copies have the probable sequence CTGQMCWFRamide (named Tcy-FRamide-1), two copies have the sequence CKGQMCWFRamide (Tcy-FRamide-2), and one copy has the sequence CVGQMCWFR-NH_2_ (Tcy-FRamide-3). It is interesting that these probable sequences contain a likely cystine bridge between the cysteine residues at positions 1 and 6 of the peptides, making them cyclic. The N-termini of the seven FRamide peptides, however, are somewhat uncertain and the proposed mature structures are based on a classical KR cleavage site preceding the sequence of the fifth copy counted from the N-terminus.
  A similar, complete preprohormone could be annotated in the transcriptome from *A. alatina* that contained six copies of an FRamide peptide (Fig. 6, Table 1). Two of these copies were identical to Tcy-FRamide-1, two other copies were identical to Tcy-FRamide-3, one copy was identical to Tcy-FRamide-2, while one copy had a new sequence CEGQMCWFRamide (Fig. 6, Table 1).
  We found an incomplete preprohormone fragment in the transcriptome from *C. fleckeri* that contained one copy of an FRamide peptide identical to Tcy-FRamide-1, and another one identical to Tcy-FRamide-2 (Fig. 6, Table 1). It is interesting that for all these proposed cubozoan FRamide peptides only the amino acid residues in position 2 were variable (being either T, K, V, or E), while the others were preserved.

**Table 1:** Annotated preprohormones and their predicted mature neuropeptide sequences

Species	Preprohormone name	Peptide name	Predicted peptide sequence	Copies
*T.cystophora*	Tcy-RFamide	RFamide-1	pQWLRGRFamide	19
		RFamide-2	pQFLRGRFamide	1
*A.alata*	Aal-RFamide	RFamide-1	pQWLRGRFamide	18
*C.xaymacana*	Cxa-RFamide	RFamide-1	pQWLRGRFamide	11
*C.quadrumanus*	Cqu-RFamide	RFamide-1	pQWLRGRFamide	1
*C.fleckeri*	Cfl-RFamide	RFamide-1	pQWLRGRFamide	7
*T.cystophora*	Tcy-RFamide-II	RFamide-II-1	RFamide	6
*A.alata*	Aal-RFamide-II	RFamide-II-1	RFamide	3
*T.cystophora*	Tcy-VWamide	VWamide-1	pQPPGVWamide	6
*A.alata*	Aal-VWamide	VWamide-1	pQPPGVWamide	6
*C.xaymacana*	Cxa-VWamide	VWamide-1	pQPPGVWamide	5
*C.fleckeri*	Cfl-VWamide	VWamide-1	pQPPGVWamide	4
		PAamide-1	pQSPAamide	1
		NWamide-1	pQGNWamide	1
*T.cystophora*	Tcy-LWamide	Peptide-1	GNPKGGSILWamide	1
		Peptide-2	pQPGMWamide	1
		Peptide-3	SLVQPRLNMLWamide	1
		Peptide-4	AMKEESPRLGLWamide	1
		Peptide-5	REMLERPKVGLWamide	1
		Peptide-6	SSKPGKVGLWamide	1
		Peptide-7	PDRPIEGLWamide	1
		Peptide-8	KGKPGTVGLWamide	1
*A.alata*	Aal-LWamide	Peptide-1	RAPRKPFILWamide	1
		Peptide-2	pQPGMWamide	1
		Peptide-3	ALVKPRLDLLWamide	1
		Peptide-4	AMVRPKLNLLWamide	1
		Peptide-5	GKMGNEPQAGLWamide	1
		Peptide-6	TSEPGKVGLWamide	1
		Peptide-7	DADAVDWLWamide	1
		Peptide-8	KPKGDAIGIWamide	1
*C.xaymacana*	Cxa-LWamide	Peptide-2	pQPGMWamide	1
		Peptide-3	ALVRPRLNLLWamide	1
		Peptide-4	ALKENGPKMGLWamide	1
*C.fleckeri*	Cfl-LWamide	Peptide-2	pQPGMWamide	1
		Peptide-3	ALVKPRLDLLWamide	1
*T.cystophora*	Tcy-RAamide	RAamide-1	RPRAamide	13
		RSamide-1	pQPRSamide	3
		RGamide-1	pQVLTRPRGamide	1
*A.alata*	Aal-RAamide	RAamide-1	RPRAamide	14
		RGamide-2	pQPRGamide	3
*C.xaymacana*	Cxa-RAamide	RAamide-1	RPRAamide	2
		RAamide-2	VPRAamide	1
*C.quadrumanus*	Cqu-RAamide	RAamide-1	RPRAamide	2
		RSamide-1	pQPRSamide	1
*C.fleckeri*	Cfl-RAamide	RAamide-1	RPRAamide	3
*T.cystophora*	Tcy-RYamide	Peptide-1	TPPWVKGRYamide	1
		Peptide-2	pQMWHRQRYamide	1
		Peptide-3	APGWHHGRYamide	1
		Peptide-4	TPLWAKGRYamide	1
*A.alata*	Aal-RYamide	Peptide-1	TPPWIKGRYamide	1
		Peptide-2	pQLWLKQRYamide	1
		Peptide-3	APGWHHGRYamide	1
		Peptide-4	GPIWFKGRYAamide	1
*C.xaymacana*	Cxa-RYamide	Peptide-3	APGWHHGRYamide	1
		Peptide-4	NPVWAKGRYamide	1
*C.fleckeri*	Cfl-RYamide	Peptide-2	pQLWYKGRYAamide	1
*T.cystophora*	Tcy-FRamide	FRamide-1	CKGQMCWFRamide	2
		FRamide-2	CTGQMCWFRamide	4
		FRamide-3	CVGQMCWFRamide	1
*A.alata*	Aal-FRamide	FRamide-1	CKGQMCWFRamide	1
		FRamide-2	CTGQMCWFRamide	2
		FRamide-3	CVGQMCWFRamide	2
		FRamide-4	CEGQMCWFRamide	1
*C.fleckeri*	Cfl-FRamide	FRamide-1	CKGQMCWFRamide	1
		FRamide-2	CTGQMCWFRamide	1

**Table 2:**
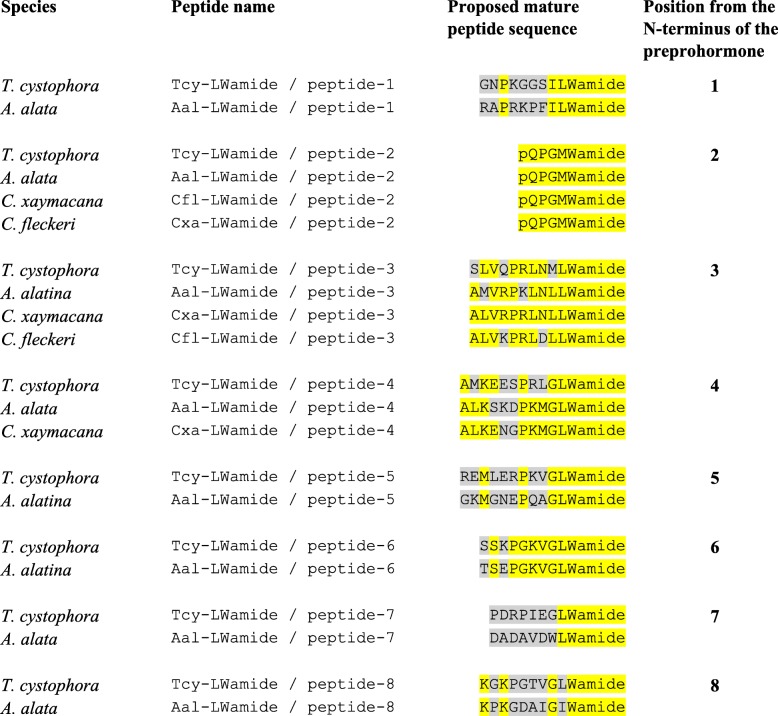
Distinct LWamide neuropeptide subfamilies located on the cubomedusan LWamide preprohormones. These neuropeptide subfamilies are located at a certain order (last column) counted from the N- to C-termini of the preprohormones. Identical amino acid residues are highlighted in yellow, non-identical residues in grey

**Table 3:**
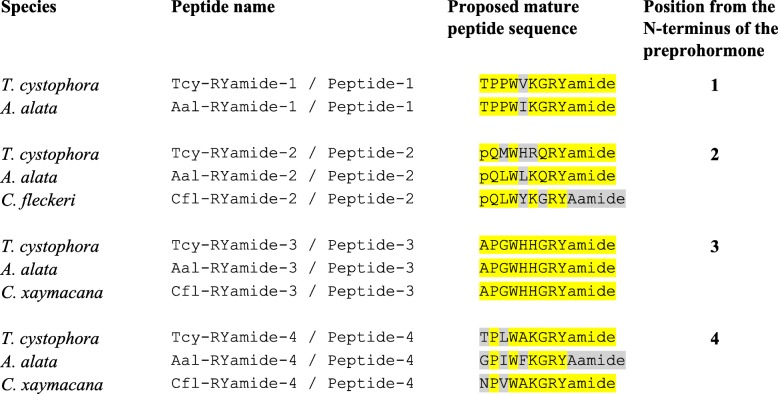
Distinct neuropeptide subfamilies located on the RYamide preprohormones from different cubomedusan species. These neuropeptide subfamilies are located at discrete positions and counted from the N- to C-termini of the preprohormones. The amino acid residues are marked as in Table 2

### Presence of glycoprotein hormone transcripts

**Fig. 7:**
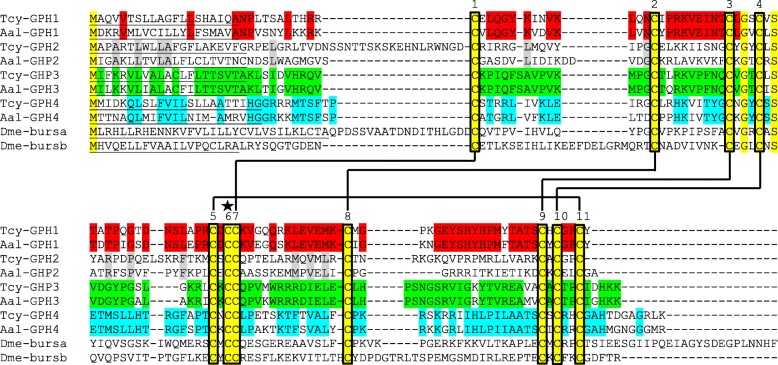
Alignment of the amino acid sequences from the glycoprotein hormones (GPHs) identified in the transcriptomes from *T. cystophora* and *A. alatina,* together with the *Drosophila* bursicon-α (Dme-bursα) and -β (Dme-bursβ) subunits*.* In *T. cystophora*, we discovered four glycoprotein hormone subunits (Tcy-GPH-1 to − 4) and the same number was found in *A. alatina* (Aal-GPH-1 to − 4). These subunits from one species can form hetero- or homodimers. Amino acid residues that were identical between two orthologues in the two cubomedusae are highlighted in the same color. Residues that are identical in all subunits are highlighted in yellow. GPHs are known to have five cystine bridges (presented as horizontal lines) formed by oxydation from ten cysteine residues (marked by vertical boxes). The star marks cysteine residue #6, which makes an intermolecular cystine bridge with the other subunit that is part of the dimer. It can be seen that the cystine bridges in the cubomedusan GPH and *Drosophila* bursicon subunits are probably the same. In addition, the cubomedusan subunits have several amino acid residues in common with the bursicon subunits, especially around cysteine residue #4. The sequences for Tcy-GPH-1 to − 4 have been submitted to the GenBank Data Bank with accession numbers MH835330-MH835333

TBLASTN screening using various mammalian and insect glycoprotein hormone sequences as a query identified four complete glycoprotein hormone subunits in our combined transcriptome database from *T. cystophora* (Fig. 7). When we applied the same procedure to the transcriptome database from *A. alata* we could identify four orthologues to the *T. cystophora* glycoprotein hormone subunits (Fig. 7). Generally, glycoprotein hormone (GPH) subunits have eleven cysteine residues, of which 10 are used for intramolecular cystine bridges, while one (number 6 in Fig. 7) is used for connecting the two subunits to form a functional ligand. Figure 7 shows that the four cubomedusan GPH subunits probably form the same cysteine bridges as the other metazoan GPHs, for example the two subunits from *Drosophila* bursicon.

### Annotation of leucine-rich repeats-containing G protein-coupled receptors (LGRs)

**Fig. 8:**
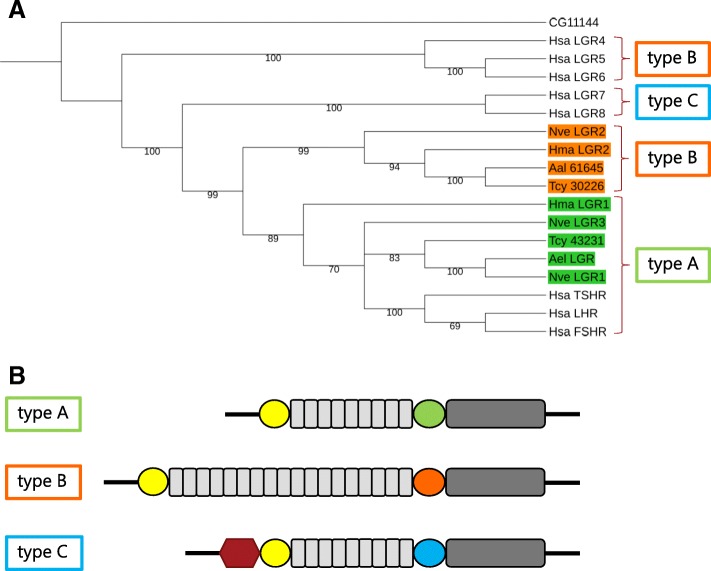
Upper panel: A phylogenetic tree analysis of the Leu-rich repeats containing G Protein-coupled receptors (LGRs) from *T. cystophora,* and *A. alata,* together with some selected LGRs from other cnidarians and humans. The tree is routed with the *Drosophila* mGlu receptor CG2114. Lower panel: A cartoon (modified from [70]), showing the characteristic features of the A-, B-, and C-type LGRs. In this cartoon the transmembrane region is highlighted in dark grey and the extracellular leucine-rich repeats in light grey (LGR types -A, -B and -C have a characteristic number of these repeats drawn as boxes). The yellow circles are cysteine-rich domains preceding the leucine-rich repeats. Type-C has a low-density lipoprotein domain (drawn as a hexagon) preceding these yellow-marked cysteine-rich domains. In between the leucine-rich repeats and the transmembrane regions are cysteine-rich domains that are specific for either the A-, B-, or C-type (given as green, orange, and blue circles) LGRs. According to these features, the *T. cystophora* Tcy-43231-LGR (the number refers to Additional file 9, “LGRs”) is an A-type LGR (highlighted in green), which clusters together with the A-type LGRs from the sea anemone *Anthopleura elegantissima* (Ael-LGR; [53]), *Hydra magnipapillata* (Hma_LGR1), and *Nematostella vectensis* (Nve-LGR1 and Nve-LGR3). The other *T. cystophora* LGR (Tcy-30226-LGR, see Additional file 9) is a B-type LGR (highlighted in orange) and clusters together with the B-type LGRs from *A. alatina* (Aal-61645-LGR, see Additional file 9), *N. vectensis* (Nve-LGR2), and *H. magnipapilla* (Hma-LGR2). Other abbreviations are: Hsa, *H. sapiens*; FSHR, follicle-stimulating-hormone receptor; LHR, luteinizing hormone receptor; TSHR, thyroid-stimulating-hormone receptor. Additional file 9 gives the GenBank Data Bank accession numbers for the cubomedusan LGRs. The accession numbers for the other LGR sequences are given in the Methods

The presence of four glycoprotein hormone subunits (yielding at least two heterodimeric glycoprotein hormone ligands) in *T. cystophora* strongly suggests the presence of Leu-rich G protein-coupled receptors (LGRs), which in mammals, insects, and other invertebrates are the receptors for glycoprotein hormones [47, 67–70]. Furthermore, already in 1993, we cloned an LGR from sea anemones [53, 54], indicating that LGRs might be present in all cnidarians. Using the sea anemone LGR and several mammalian and insect LGRs as queries in a TBLASTN search, we were able to identify two LGR transcripts in the database from *T. cystophora* and one LGR in the transcriptome from *A. alata* (Table 4, Fig. 8, Additional file 9). Figure 8b explains that LGRs can be classified into three types, type-A, -B, and -C, depending on specific domains in their extracellular N-termini [70]. These criteria identify the two *T. cystophora* LGRs as being type-A and -B, respectively (Fig. 8a). The single LGR from *A. alata* is a type-B LGR and an orthologue of the type-B LGR from *T. cystophora*. We assume that the absence of the A-type LGR family member in *A. alata* is due to insufficient coverage of the assembled transcriptome from this cubomedusa.

**Table 4:** Overview of the numbers of GPCRs identified in the transcriptomes from *T. cystophora* and *A. alata*

Type of GPCR	Number in *T. cystophora*	Number in *A. alata*
Presumed biogenic amine receptors	28	16
Presumed neuropeptide receptors	22	18
Opsins	5	2
LGRs	2	1

### Presence of opsins

**Fig. 9:**
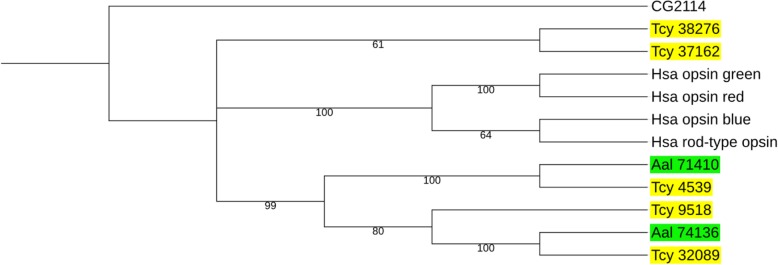
Phylogenetic tree analysis of four human opsins together with the five opsins identified in the transcriptome from *T. cystophora* (marked in yellow) and the two opsins from *A. alata* (marked in green). The tree has been rooted with the *Drosophila* mGlu receptor CG2114. The numbers given after the abbreviations Tcy and Aal refer to the ones given in Additional file 9. The *T. cystophora* opsin Tcy32089 is expressed in the lense-eye, supporting that it is a functional opsin [73]. It can be seen that two opsins from *T. cystophora* have close orthologues in *A. alata*. See additional file 9 for GenBank Data Bank accession numbers of the cubomedusan opsins, and Methods for the accession numbers of the human opsins

It is known that cubomedusae and other cnidarians produce opsins [13, 61, 71–75]. We used cnidarian, and other invertebrate and vertebrate opsins as queries in a TBLASTN search of our *T. cystophora* transcriptome and found five different opsins (Fig. 9 and Table 4). A similar screening of the *A. alata* transcriptome yielded two opsins, which were orthologues of two of the *T. cystophora* opsins (Fig. 9).

### Presence of neuropeptide and biogenic amine GPCRs

**Fig. 10:**
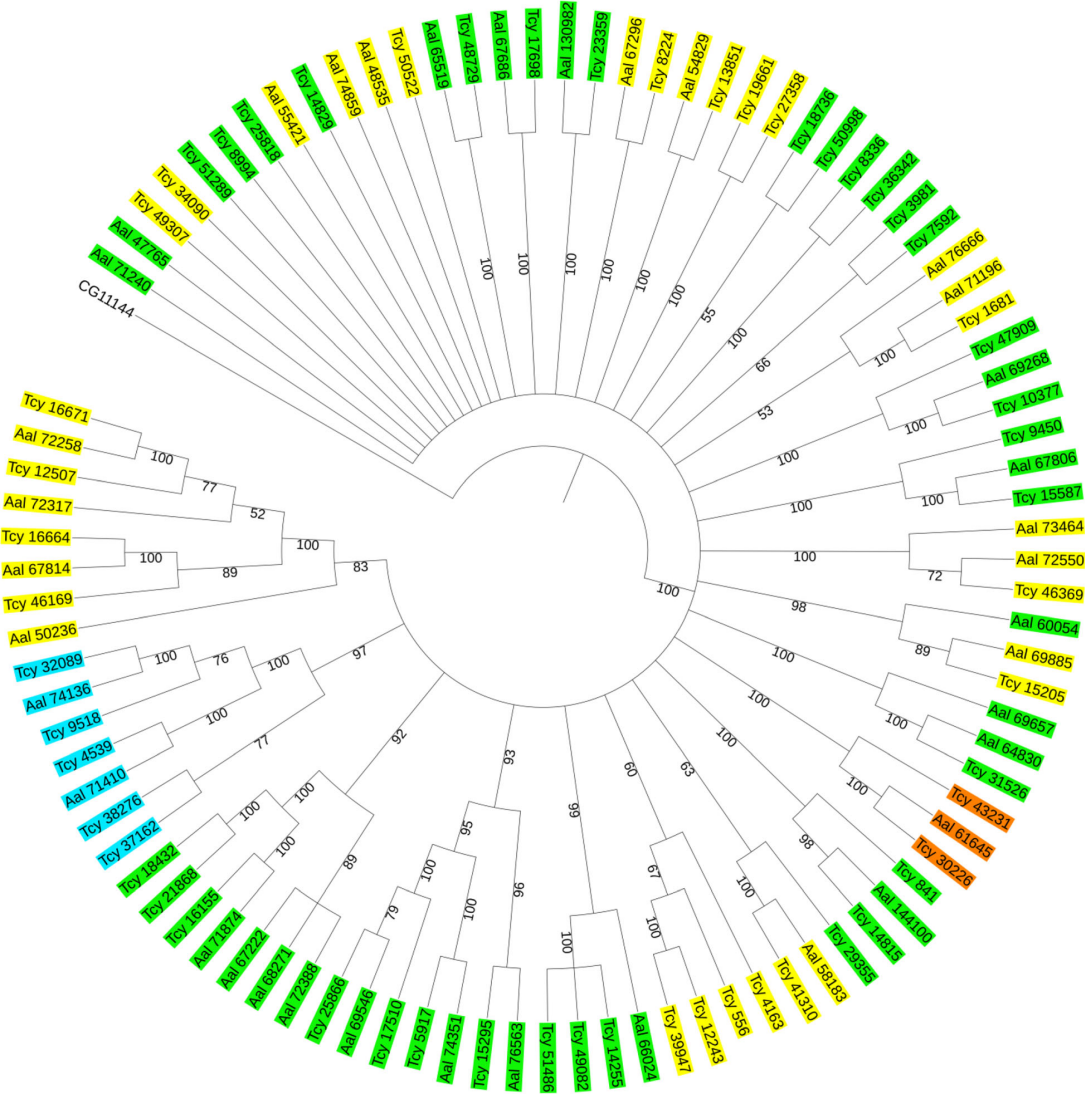
Phylogenetic tree analysis of the family-A (rhodopsin-like) GPCRs discovered in the transcriptomes from *T. cystophora* and *A. alata.* The tree has been rooted with the *Drosophila* mGlu receptor CG2114. The opsins are highlighted in blue. The LGRs are highlighted in orange. When, during a TBLASTN search, the receptor was computer-identified as a neuropeptide GPCR, the cubomedusan GPCR was highlighted in yellow in this figure. Computer-identification as a biogenic amine GPCR let us to highlight these receptors in green. It can be seen that the computer-identified neuropeptide and biogenic amine GPCRs do not form two discrete clusters and, therefore, it is difficult to conclude that a certain receptor is a neuropeptide or biogenic amine receptor. See also Additional file 9 for GenBank Data Bank accession numbers

We used all *Drosophila* neuropeptide and biogenic amine GPCRs [47] as queries in TBLASTN searches of our *T. cystophora* transcriptome. In this way, we identified 22 GPCRs, which the TBLASTN search software described as neuropeptide GPCR-like and 28 GPCRs which the search software described as biogenic amine GPCR-like (Table 4). A similar TBLASTN search of the *A. alatina* transcriptome identified 22 neuropeptide GPCR-like receptors and 18 biogenic amine GPCR-like receptors. When we carried out a phylogenetic tree analysis of these receptors together with the *T. cystophora* and *A. alata* opsins and LGRs (see above), we found that the opsins and LGRs were sorted as discrete, structurally related clusters (Fig. 10). For the receptors that the TBLASTN software identified as neuropeptide or biogenic amine GPCRs, however, no such homogeneous clustering could be observed and all receptors were distributed randomly (Fig. 10). These results make it difficult to predict whether a certain GPCR is a neuropeptide or biogenic amine receptor.

## Discussion

In this article we described a high-quality transcriptome database from the cubomedusa *T. cystophora*, which was constructed by the combined use of Illumina and PacBio sequences, and which we made freely accessible to global researchers (NCBI accession numbers SRR7791343-SRR7791345 and GGWE01000000). The longer PacBio sequences were needed for the correct assembly of the shorter Illumina sequences, especially when these Illumina sequences coded for neuropeptide preprohormones, which often contained repetitive sequences (see, for example, Table 1). The PacBio sequences were also needed for the correct annotations of full length GPCRs (Figs. 8, 9, and 10). The Illumina sequences, on the other hand, were necessary to correct for point mutations in the PacBio sequences. In addition, we developed a bioinformatics tool to search the transcriptome database for the presence of neuropeptide preprohormones, which turned out to be a highly versatile script and superior to ordinary TBLASTN searches, using neuropeptide sequences from bilaterian metazoans as queries. Finally, we tested the transcriptome database for its quality and completeness by annotating several components of peptidergic signaling and by comparing these results from our transcriptome with those from other freely accessible transcriptomes from cubozoans [65, 76, 77]. We identified the same number of neuropeptide preprohormone genes (seven) in the transcriptomes from *T. cystophora,* and *A. alata*, while we found six neuropeptide genes in the *C. fleckeri* transcriptome, five neuropeptide genes in the *C. xaymacana* transcriptome, and two neuropeptide genes in the *C. quadrumanus* transcriptome (Table 1, Figs. 1, 2, 3, 4, 5 and 6). In most cases only incomplete preprohormone fragments could be identified in the transcriptomes from *C. fleckeri, C. xaymacana,* and *C. quadrumanus*, while always complete preprohormones (including a signal peptide and a stop codon) were identified in the transcriptome from *T. cystophora,* and with one exception in the transcriptome from *A. alata* (Figs. 1, 2, 3, 4, 5 and 6). These findings already suggest that the transcriptomes from *T. cystophora* and *A. alata* [65] are of much better quality (more complete) than the transcriptomes from *C. fleckeri, C. xaymacana,* and *C. quadrumanus* [76, 77].

When we annotated GPCRs, we discovered 50 neuropeptide and biogenic amine GPCRs in the transcriptome from *T. cystophora* and 34 of these GPCRs in the transcriptome from *A. alata* (Table 4). For the LGRs, these numbers were two in the *T. cystophora* transcriptome and one in the *A. alata* transcriptome. For the opsins, we found five in the *T. cystophora* transcriptome and two in the *A. alata* transcriptome. These somewhat lower numbers of annotated GPCRs in the *A. alata* transcriptome [65] might be due to the fact that this transcriptome is only assembled from short Illumina transcripts, while our *T. cystophora* transcriptome also contains a large number of long PacBio transcripts with a length of up to 5000 bp (Additional file 2A), which would favor the detection of longer proteins, such as GPCRs.

The number of opsins (five) that we found in our transcriptome is lower than the number of opsin genes (eighteen: Tcop1-Tcop18) claimed by Liegertova et al. [74] to be present in the genome from *T. cystophora*. However, this last claim cannot be checked, because the genomic sequences from *T. cystophora* have not been made publicly available [74]. Our identified opsin Tcy 38276 (Fig. 9) is identical to the *Tripedalia* c-opsin cloned previously [75] and opsin Tc-neo [73]; it corresponds to the opsin gene Tcop18 [74]. Our opsin Tcy 32089 (Fig. 9) was cloned previously as the lens eye opsin Tc-leo [73] and corresponds to the opsin gene Tcop13 [74]. Tcy 9518 and Tcy 4539 (Fig. 9) correspond to the opsin genes Tcop5 and Tcop9, respectively [74]. The last of the five opsins that we identified, Tcy 37162 (Fig. 9), is new.

Some of the neuropeptides that we predicted from the seven *T. cystophora* preprohormone cDNAs (Tables 1, 2 and 3) are identical or very similar to earlier chemically isolated and sequenced cnidarian neuropeptides. The Tcy-RFamide preprohormone (Table 1), for example, contains 19 copies of the predicted neuropeptide sequence pQWLRGRFamide (Tcy-RFamide-1), which is identical to the isolated and sequenced scyphozoan neuropeptide Cyanea-RFamide-I and very similar to the hydrozoan neuropeptides Pol-RFamide-II and Hydra-RFamide-I (Table 5). The C-terminal GRFamide sequence has been found in isolated or cloned neuropeptides from every cnidarian species investigated so far and the GRFamide neuropeptide family, therefore, appears to be universal in Cnidaria. The Tcy-VWamide preprohormone (Table 1) produces six copies of the predicted neuropeptide pQPPGVWamide (Tcy-VWamide-1), which resembles a previously isolated sea anemone neuropeptide metamorphosin-A [19], and the *Hydra* neuropeptide Hym331/Hydra-LWamide-I [32] (Table 6). Also, peptides belonging to the Tcy-LWamide preprohormone, such as peptides − 4, − 5, and − 6 (Tables 1 and 2) clearly resemble metamorphosin-A, with which they have the C-terminal sequence GLWamide in common (Table 6). Preprohormones containing GLWamide peptides have recently been identified in the transcriptomes from the hydrozoans *Clythia hemispheria* and *Cladonema pacificum* [33]. Thus, like the GRFamides, also GLWamide neuropeptides appear to be widespread in cnidarians.

**Table 5:**
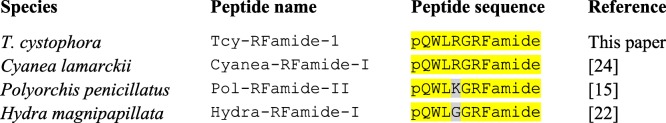
Amino acid residue identities (highlighted in yellow) between Tcy-RFamide-1 and some chemically isolated and sequenced (“established”) cnidarian neuropeptides

**Table 6:**
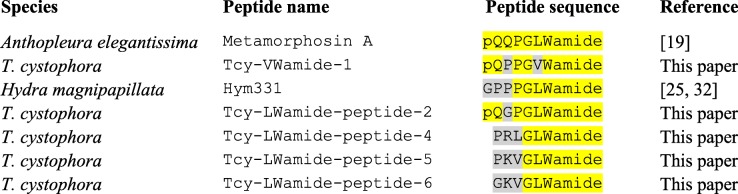
Amino acid residue identities (highlighted in yellow) between the isolated and sequenced sea anemone neuropeptide metamorphosin-A, the *Hydra* peptide Hym331, and some of the *T. cystophora* peptides contained in the Tcy-VWamide and Tcy-LWamide preprohormones (see Table 1)

The Tcy-RAamide preprohormone contains 13 copies of RPRAamide (Table 1). RPRAamide peptides and a corresponding preprohormone have recently been identified in the transcriptome from the hydrozoan *C. pacificum* [33], suggesting that also these neuropeptides might have a broad distribution.

The last two preprohormones presented in Table 1, Tcy-RYamide and Tcy-FRamide (see also Fig. 5, and Fig. 6), are completely novel sequences and also their neuropeptide constituents have not been published earlier. These results show that our *T. cystophora* transcriptome contains novel and highly useful data for understanding neurotransmission in cubozoa and possibly also in other cnidarians.

As a next practical step, we will raise specific antisera against the major neuropeptides produced by the seven preprohormones (Table 1) and clarify which neuronal subpopulations can be stained by them. These experiments will certainly give us important information on the neuroanatomy of *T. cystophora* and will also tell us which of these peptidergic nerve nets will innervate the eyes.

In conclusion, we are presenting a high-quality transcriptome from *T. cystophora*, which will be a useful resource for the scientific community to better understand the biology of early metazoans and the evolution of important tissues and organs, such as nervous systems and eyes.

## Methods

### *T. cystophora* culture and collection

*T. cystophora* (Conant 1897) were cultured in 250 l tanks with recycled sea water at 28 °C and fed with *Artemia salina* once a day. The light: dark cycle was 8:16 h. We sampled medusae of various stages with a bell diameter ranging between 3.5 and 9 mm. A total of 12 medusae were collected after 48 h of starvation.

### RNA extraction

Total RNA was extracted using the NucleoSpin RNAIIkit (Macherey-Nagel, Düren, Germany) following the manufacture’s instruction. Total RNA was dissolved in RNase-free water and RNA integrity was verified by gel electrophoresis. The RNA concentration and purity was measured with a Nanodrop ND-2000 spectrophometer (NanoDrop products, Wilmington, DE, USA).

### cDNA library construction

Total RNA samples were shipped on dry ice to an affiliation of the Beijing Genome Institute (BGI Tech Solutions, Hong Kong) for library preparation, sequencing, and bioinformatic analysis (coordinated by BGI Tech Solutions, Shenzhen, China). Sample quality and RNA concentrations were checked using the Agilent model 2100 Bioanalyzer (Agilent Technologies, Santa Clara, CA, USA) and approved for sequencing (RNA Integrity Number, RIN: 9.7, and 28S/18S: 1.7). 8.6 microgram RNA was used to construct two cDNA libraries separately. The PacBio Iso-Seq libraries with a size of 0-5 kb (Pacific Biosciences, Menlo Park, CA, USA) were generated for sequencing on two SMRT cells and one RNA-Seq library was prepared for sequencing with Illumina X Ten (Illumina, San Diego, CA, USA).

### PacBio Iso-Seq de novo assembly and read correction

Additional file 4A and B give an overview of the PacBio sequencing procedures and data processing. The bioinformatic data processing and error corrections were conducted at Beijing Genome Institute (BGI Tech Solutions, Shenzhen, China) following the PacBio Iso-Seq De novo protocol. The raw reads generated from the SMRT (Single Molecule Real Time) pipeline were separated into FL (full length) non-chimeric, non-FL and chimeric ROI. The chimerae, which were artificial contatemers fusion genes, were removed by this step. The FL non-chimeric ROI’s were defined as having the 5′ prime, 3′ prime-adapters and a polyA tail. The FL non-chimeric ROI’s were assembled to generate transcripts of all FL non-chimeric and non-FL non-chimeric sequences. For each assembled transcript, the Quiver error self-correction (polishing) software was run [78]. These corrected transcripts were divided into a high quality (hq; expected accuracy ≥99%, or QV ≥ 30) and a low quality (lq; expected accuracy < 99%, due to insufficient coverage or rare transcripts) subset. Even though the error rate was reduced by this procedure, a further correction was performed using Illumina RNA-Seq reads from the same sample (see below) and two additional bioinformatic packages, proovread [62] and Long Read de Bruijn Graph Error Correction (LoRDEC) [63]. Default parameters applied in proovread were -t 5 -b 200 -e 0.4 -s 3 -T 4 and k-mers 21 and 25 were used in LoRDEC. For details on the bioinformatic pipeline see Additional file 4.

### Illumina RNA-Seq data processing

Illumina sequencing was performed with the Illumina X Ten machine using standard procedures and FastQC tools [79, 80]. Raw reads were subjected to quality filtration [81]. The filtering procedure performed to obtain “clean reads” with a high quality score, included the following steps: 1) Reads with adaptor sequences were removed 2) Reads in which the percentage of unknown bases (N) > 10% were removed 3) Low quality reads consisting of more than 40% low quality bases (value ≤5) and having a Phred score less than 20, were also removed.

### Identification of neuropeptides, protein hormones, and GPCRs

We developed a software program to identify putative neuropeptides (Additional files 6 and 7). This program was compiled using Python3 [82]. Our software was based on recognizing and counting prohormone convertase processing sites in the amino acid sequence. Because many mature neuropeptides are amidated, the preprohormone often contains a C-terminal glycine before the basic amino acid processing sites. This was accounted for in the searches (e.g. searching for ‘GKR’, ‘GKK’ and ‘GR’ motifs). For each sequence from the TSA (transcriptome shotgun assembly), the sequence was translated into all 6 reading frames and split into possible open reading frames. In all of the open reading frames, the number of processing sites was counted. If there were at least 3 processing sites within an open reading frame, the putative neuropeptide sequences were aligned to assess the similarity of the mature peptides. The open reading frames with highly similar peptide sequences were then manually curated to reject further unlikely preprohormones that were not discarded during the automated screening. The flow chart of the program can be seen in Additional file 6. The code can be found at [64] and in Additional file 7. The presence of signal peptides was determined by SignalP 4.1 [83, 84]. We identified glycoprotein hormones and GPCRs in the *T.cystophora* and *A.alata* transcriptomes by homology based searches. These searches were done with known cnidarian and other invertebrate and vertebrate protein sequences as search queries using TBLASTN [85] with default settings.

### Phylogenetic tree analyses and accession numbers

For phylogenetic tree analyses (Figs. 8, 9, 10), the protein sequences were aligned using t-coffee. The alignments were read and analyzed in PAUP* by neighbor joining using p-distance and bootstraps of 100 repeats. The majority rule consensus trees were visualized using iTOL. Only bootstrap values above 50 were given in the figures. For Fig. 7, the following accession numbers for the *Drosophila* sequences were used: Dmel-bursα, NP_650983; Dmel-bursβ, NP_609712. For Fig. 8, the following accession numbers were used: Hma LGR1, XP_002155960; Hma LGR2, NP_001267732; Ael LGR, CAA82186; Nve LGR1, XP_001641580; Nve LGR2, XP_001635321; Nve LGR3, XP_001638153; Hsa FSHR, NP_000136; Hsa TSHR, NP_000360; Hsa LHR, NP_000224; Hsa LGR4, NP_060960; Hsa LGR5, NP_003658; Hsa LGR6, NP_001017403; Hsa LGR7, NP_067647; Hsa LGR8, AAL69324. For Fig. 9, Hsa opsin-blue, NP_001699; Hsa opsin-red, NP_064445; Hsa opsin-green, NP_000504; Hsa-rhodopsin, NP_000530.

## Additional files

Additional file 1: A: Quality assessment of PacBio data. B: Read length distribution of ROIs from the first PacBio sequencing round. C. Read length classification summary of the first PacBio sequencing round. D: PacBio output summary from the first PacBio sequencing round. (DOCX 76 kb)

Additional file 2: A: Read length distribution of all ROIs from combined data from the first and second PacBio sequencing rounds. B: Read length classification summary of the combined data from the first and second sequencing rounds. C: PacBio output summary of the combined PacBio data from the first and second sequencing rounds. (DOCX 61 kb)

Additional file 3: Illumina HiSeq X Ten pipeline output summary. (DOCX 17 kb)

Additional file 4: A: PacBio Iso-Seq data processing and read correction. B: PacBio IsoSeq data processing pipeline illustrated. (DOCX 150 kb)

Additional file 5: Comparison of the transcripts from the *T. cystophora* transcriptome with those from selected other eukaryotes, including a Venn diagram. (DOCX 1883 kb)

Additional file 6: A flow diagram of our software used to predict neuropeptide preprohormones in a transcriptome. (DOCX 137 kb)

Additional file 7: Our script written in Python3 used to predict neuropeptide preprohormones in a transcriptome. (PY 3 kb)

Additional file 8: The amino acid sequences of the predicted RFamideII preprohormones from *T. cystophora* and *A. alata. (DOCX 20 kb)*

Additional file 9: A table, including accession numbers of biogenic amine GPCRs, neuropeptide GPCRs, LGRs, and opsins present in the transcriptomes from *T. cystophora* and *A. alata. (XLSX 13 kb)*

## Abbreviations

Aal: *Alatina alata*
Ael: *Anthopleura elegantissama*
BGI: Beijing Genome Institute
Burs: Bursicon
Cfl: *Chironex fleckeri*
Cqu: *Chiropsalmus quadrumanus*
Cxa: *Carybdea xaymacana*
DEG/ENaC: Degenerin/epithelial Na^+^ channel
Dme-burs: *Drosophila melanogaster* bursicon.
DPAP: Dipeptidyl aminopeptidase
FL: Full length
FSH: Follicle-stimulating-hormone
GPCR: G protein-coupled receptor
GPH: Glycoprotein hormone
Hma: *Hydra magnipapillata*
hq: High quality
Hsa: *Homo sapiens*
ICE: Iterative clustering for error correction
LGR: Leucine-rich repeats containing G protein-coupled receptor
LH: Luteinizing hormone
LoRDEC: Long read de Bruijn graph error correction
lq: Low quality
Nve: *Nematostella vectensis*
PacBio: Pacific Biosciences
PC: Prohormone convertase
pQ: Pyroglutamate residue (cyclized glutamine residue)
ROI: Reads of insert
SMRT: Single molecule real time
SRA: Sequence read archive
Tcy: *Tripedalia cystophora*
TRH: Throtropin-releasing-hormone
TSA: Transcriptome shotgun assembly
TSH: Thyroid-stimulating-hormone
