# Supplementary material for: De novo transcriptome assembly of the cubomedusa Tripedalia cystophora, including the analysis of a set of genes involved in peptidergic neurotransmission
Source: BMC Genomics. 2019 Mar 6;20:175. doi: 10.1186/s12864-019-5514-7 (PMC6402141; doi:10.1186/s12864-019-5514-7)
Supplement: Supplementary file 1 — A: Quality assessment of PacBio data. B: Read length distribution of ROIs from the first PacBio sequencing round. C. Read length classification summary of the first PacBio sequencing round. D: PacBio output summary from the first PacBio sequencing round. (DOCX 76 kb) [file 12864_2019_5514_MOESM1_ESM.docx]

**Additional File 1 A**

**Quality Assesment of PacBio Data**

**Reads of Insert (ROI) Quality Index**
Read quality reflects the correct identification of the sequenced base. It is therefore a measure for base accuracy and its maximum is 1.0.

**Transcript Quality**Transcript quality is based on read coverage (number of reads/transcript) and read quality (see above)

Additional file 1. Length distribution of all ROI data – from 1 SMRT cell run.

**Additional File 1 B**

**Read length distribution of ROI from first PacBio sequencing round**


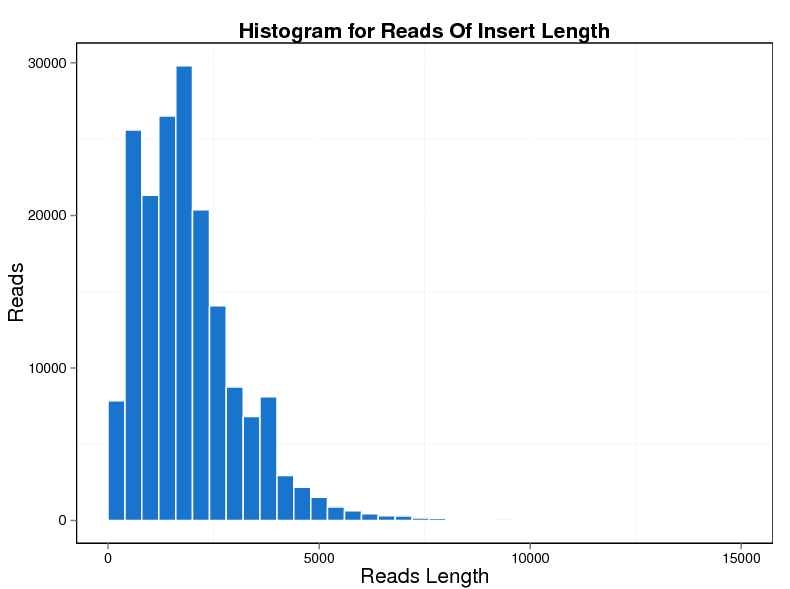


**Additional File 1 C**


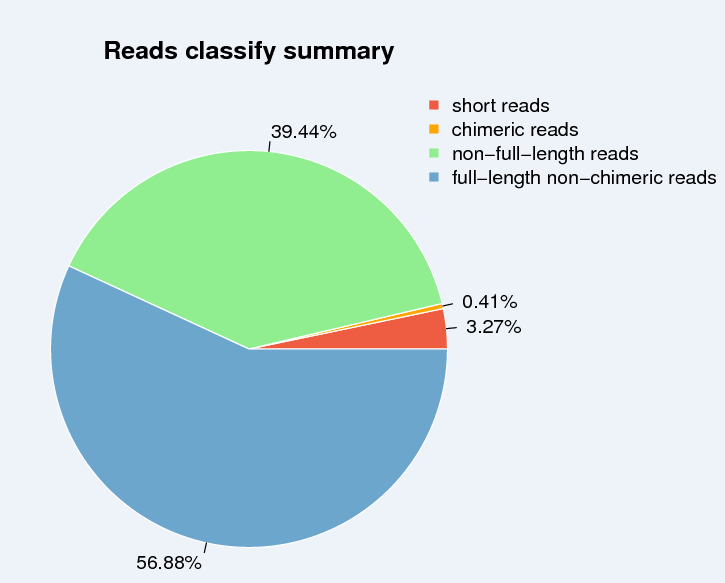
**Read length classification summary of first PacBio sequencing round**

Pie chart of reads of insert (ROI) classification.

**Additional File 1 D**

**PacBio output summary from first PacBio sequencing round**

| Data Type PacBio Iso-Seq – Data Set from single SMRT cell | Library size 0-5 kb |
| --- | --- |
| Number of reads of Insert (ROI)  Number of five prime reads  Number of three prime reads  Number of poly-A reads  Number of filtered short reads (threshold: < 300bp)  Number of Chimeric reads  Number of full-length non chimeric reads  Number of non-full-length non chimeric reads  Number of polished high-quality isoforms  Number of polished low-quality isoforms  Number of unique transcripts (consensus isoforms)  Average transcript read length (bp) N50(bp)^[[1]](#footnote-1)^ of unique transcripts | 179.249 137.352 (76.63 %) 156.510 (87.31 %) 114.630(63.95 %) (3.27 %)  (0.41%) ^[[2]](#footnote-2)^ 101.955 (56.88 %)  70.676 (39.44 %) 38.731 34.652 **25.779** (38.731)^[[3]](#footnote-3)^ 1.893 bp 2.280 bp |

1. N50 define assembly quality in terms of contiguity. N50 is a weighted median statistics which define 50% of the entire assembly to be contained in transcripts equal to or larger than this defined length. [↑](#footnote-ref-1)
2. The number of artificial contatemers is very low. This indicates a successful SMRTbell library prep [↑](#footnote-ref-2)
3. Number in bold is the number of unique transcripts after redundancy are removed. The number in the paragraph is the merged number of transcripts. [↑](#footnote-ref-3)
