# Supplementary material for: De novo transcriptome assembly of the cubomedusa Tripedalia cystophora, including the analysis of a set of genes involved in peptidergic neurotransmission
Source: BMC Genomics. 2019 Mar 6;20:175. doi: 10.1186/s12864-019-5514-7 (PMC6402141; doi:10.1186/s12864-019-5514-7)
Supplement: Supplementary file 2 — A: Read length distribution of all ROIs from combined data from the first and second PacBio sequencing rounds. B: Read length classification summary of the combined data from the first and second sequencing rounds. C: PacBio output summary of the combined PacBio data from the first and second sequencing rounds. (DOCX 61 kb) [file 12864_2019_5514_MOESM2_ESM.docx]

**Additional File 2 A**

**Read length distribution of all ROI from combined data from first and second PacBio sequencing round**


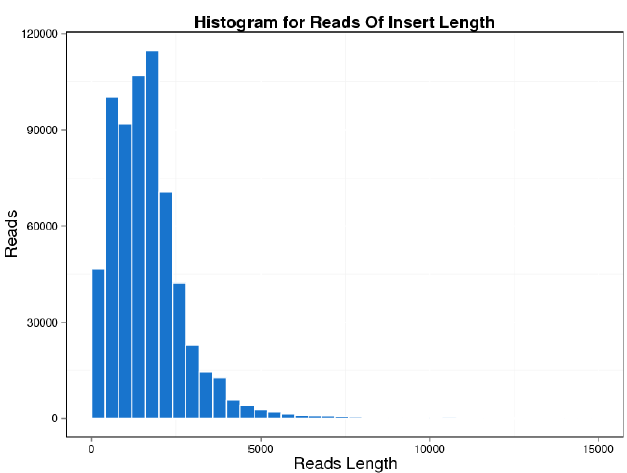


**Additional File 2 B**

**Read length classification summary of combined PacBio data from first and second sequencing round**


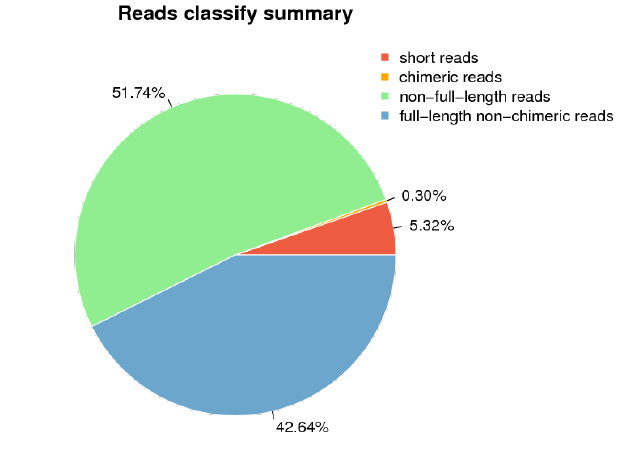


**Additional File 2 C**

**PacBio output summary of combined PacBio data from first and second sequencing round**

| Data Type PacBio Iso-Seq – Merged Data Set  from 2 SMRT cells | Library size 0-5 kb |
| --- | --- |
| Number of reads of Insert (ROI)  Number of five prime reads  Number of three prime reads  Number of poly-A reads  Number of filtered short reads (threshold: < 300bp)  Number of Chimeric reads  Number of full-length non chimeric reads  Number of non-full-length non chimeric reads  Number of polished high-quality isoforms  Number of polished low-quality isoforms  Number of unique transcripts (consensus isoforms) Average transcript read length (bp) N50(bp)^[[1]](#footnote-1)^ of unique transcripts | 645.865 374.698 (58.01%)  463.988 (71.84%)  336.758(52.14%) (5.32%)  (0.30%) ^[[2]](#footnote-2)^ 275.377 (42.64%)  334.235 (51.74%)  88.588 106.394  **46.348** (88.558)^[[3]](#footnote-3)^ 1.682 bp 1992 bp |

1. N50 define assembly quality in terms of contiguity. N50 is a weighted median statistics which define 50% of the entire assembly to be contained in transcripts equal to or larger than this defined length. [↑](#footnote-ref-1)
2. The number of artificial contatemers is very low. This indicates a successful SMRTbell library prep. [↑](#footnote-ref-2)
3. Number in bold is the number of unique transcripts after redundancy are removed. The number in the paragraph is the merged number of transcripts. [↑](#footnote-ref-3)
