# Supplementary material for: De novo transcriptome assembly of the cubomedusa Tripedalia cystophora, including the analysis of a set of genes involved in peptidergic neurotransmission
Source: BMC Genomics. 2019 Mar 6;20:175. doi: 10.1186/s12864-019-5514-7 (PMC6402141; doi:10.1186/s12864-019-5514-7)
Supplement: Supplementary file 3 — Illumina HiSeq X Ten pipeline output summary. (DOCX 17 kb) [file 12864_2019_5514_MOESM3_ESM.docx]

**Additional File 3**

| Data Type Illumina PE | Raw Data | Clean Data |
| --- | --- | --- |
| Number of reads  Data size  Number of fastq 1. read  Number of fastq 2. read  GC% content fastq 1. read  GC% content fastq 2. read  Q20% fastq 1. read  Q20% fastq 2. read  Q30% fastq 1. read  Q30% fastq 2. read  Error ratio (%) fastq 1. read  Error ratio (%) fastq 2. read  Discarded reads related to N^[[1]](#footnote-1)^  Discarded reads related to low quality^[[2]](#footnote-2)^  Discarded reads related to adaptor | 223 Million  33.4 Gbyte  244730  254564 46.19 46.15  98.60  97.65  96,68  94.89  16.80  18.82  672  10419608  8899414 | 203 Million  30.5 Gbyte  210505 223856  46.14  46.08 99.45  99.14  98.18  97.24  0.08  0.12 |

**Illumina HiSeq X Ten pipeline output summary**

1. Removed reads in which unknown bases(N) are more than 5% [↑](#footnote-ref-1)
2. Low quality reads are defined as reads where the percentage of low quality (phred score) bases is >20%. A low quality base is defined as one whose sequencing quality is less than 15. [↑](#footnote-ref-2)
