# Supplementary material for: De novo transcriptome assembly of the cubomedusa Tripedalia cystophora, including the analysis of a set of genes involved in peptidergic neurotransmission
Source: BMC Genomics. 2019 Mar 6;20:175. doi: 10.1186/s12864-019-5514-7 (PMC6402141; doi:10.1186/s12864-019-5514-7)
Supplement: Supplementary file 4 — A: PacBio Iso-Seq data processing and read correction. B: PacBio IsoSeq data processing pipeline illustrated. (DOCX 150 kb) [file 12864_2019_5514_MOESM4_ESM.docx]

**Additional File**
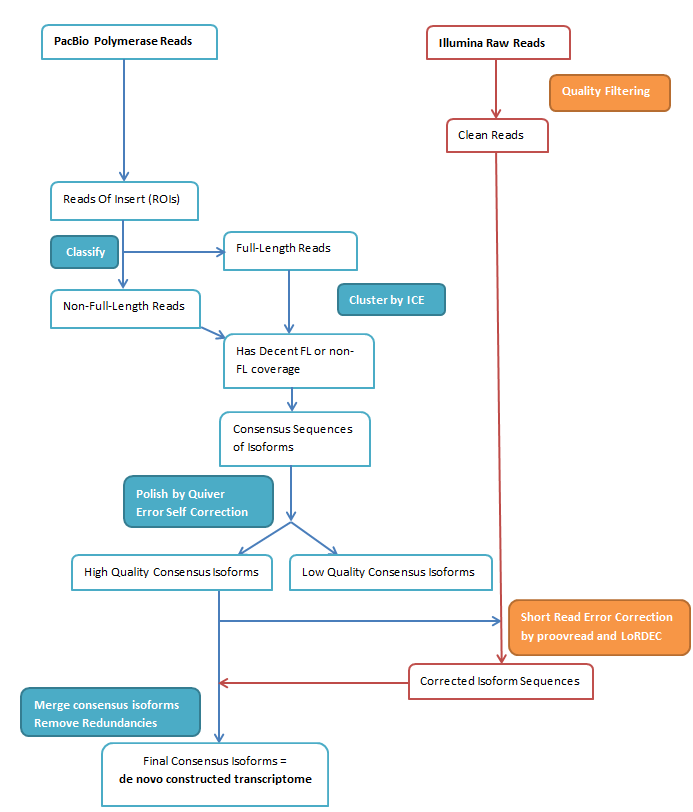
**4 A**

**PacBio Iso-Seq data processing and read correction**

**Additional File 4 B**

**PacBio Iso-Seq data processing pipeline illustrated**


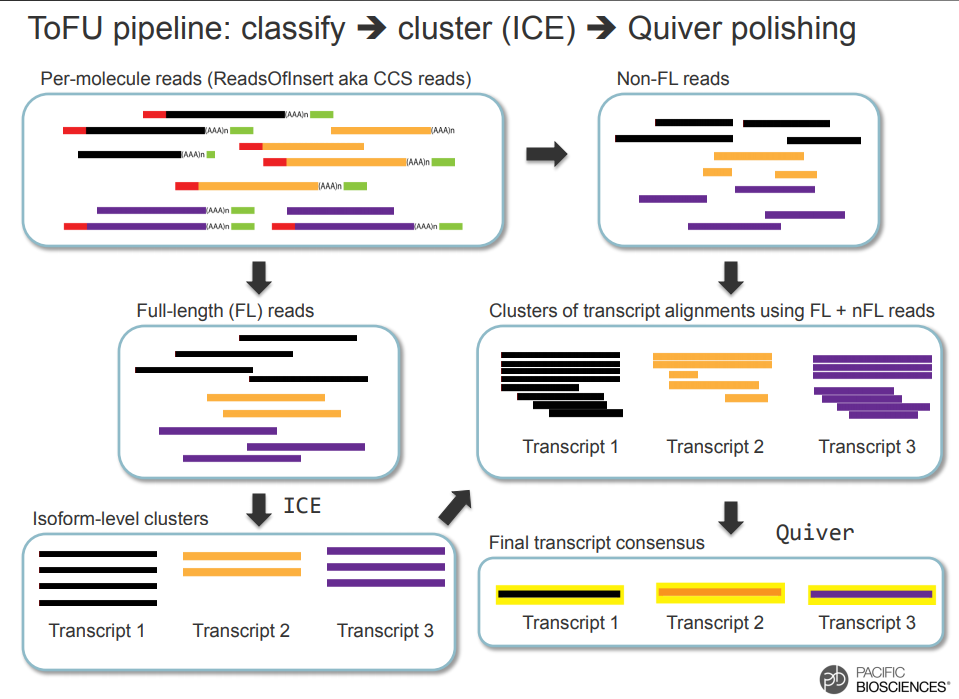


Illustration of **PacBio Iso-Seq data processing** (adopted from Pacific Biosciences) following the pipeline: Classify → Cluster (ICE) → Polish (Quiver) (merge and remove redundancy) = Final unique transcripts
