# Supplementary material for: De novo transcriptome assembly of the cubomedusa Tripedalia cystophora, including the analysis of a set of genes involved in peptidergic neurotransmission
Source: BMC Genomics. 2019 Mar 6;20:175. doi: 10.1186/s12864-019-5514-7 (PMC6402141; doi:10.1186/s12864-019-5514-7)
Supplement: Supplementary file 5 — Comparison of the transcripts from the T. cystophora transcriptome with those from selected other eukaryotes, including a Venn diagram. (DOCX 1883 kb) [file 12864_2019_5514_MOESM5_ESM.docx]

Additional file 5. Functional annotations of the T. cystophora transcripts


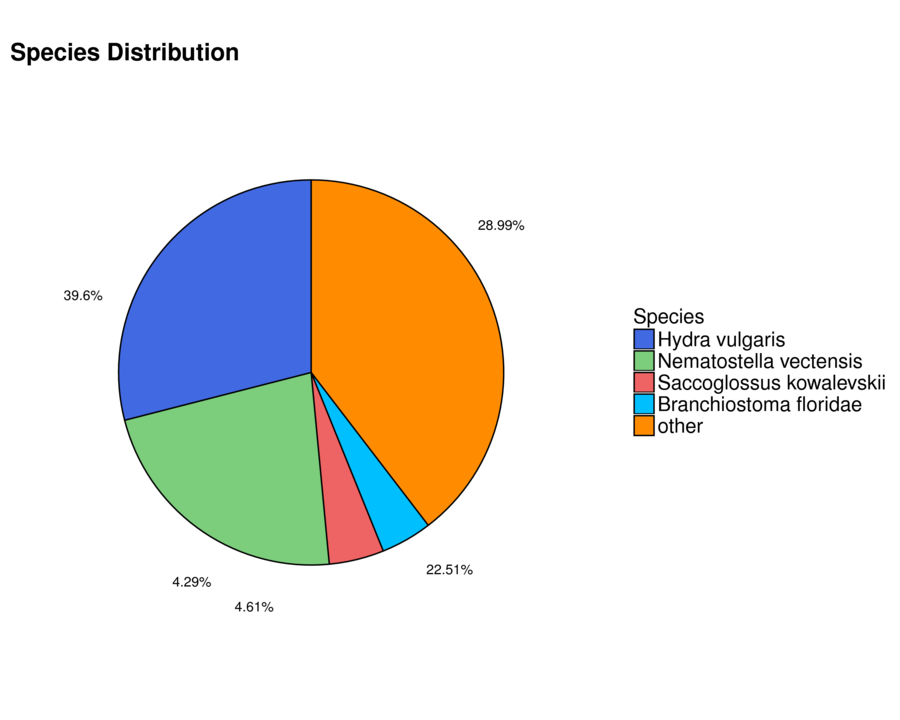


**File 5A**, Distribution of non-redundant annotated orthologues in other invertebrate species


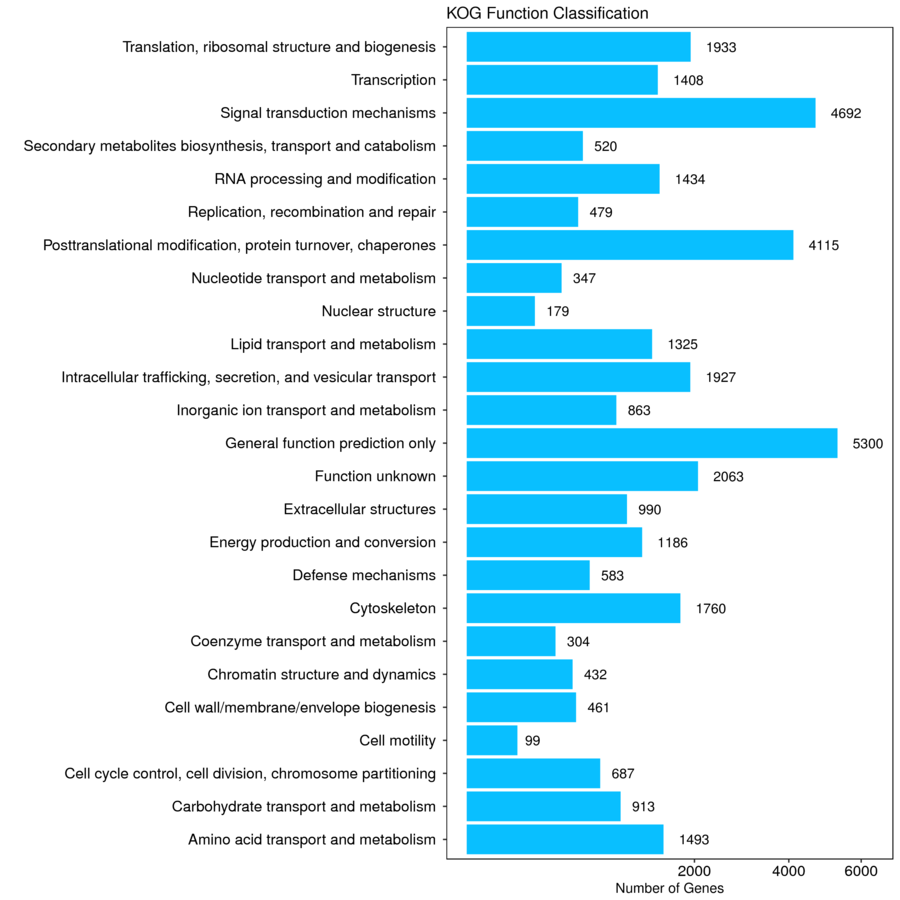


**File 5B**, Annotations of functional groups present in the T. cystophora transcriptome


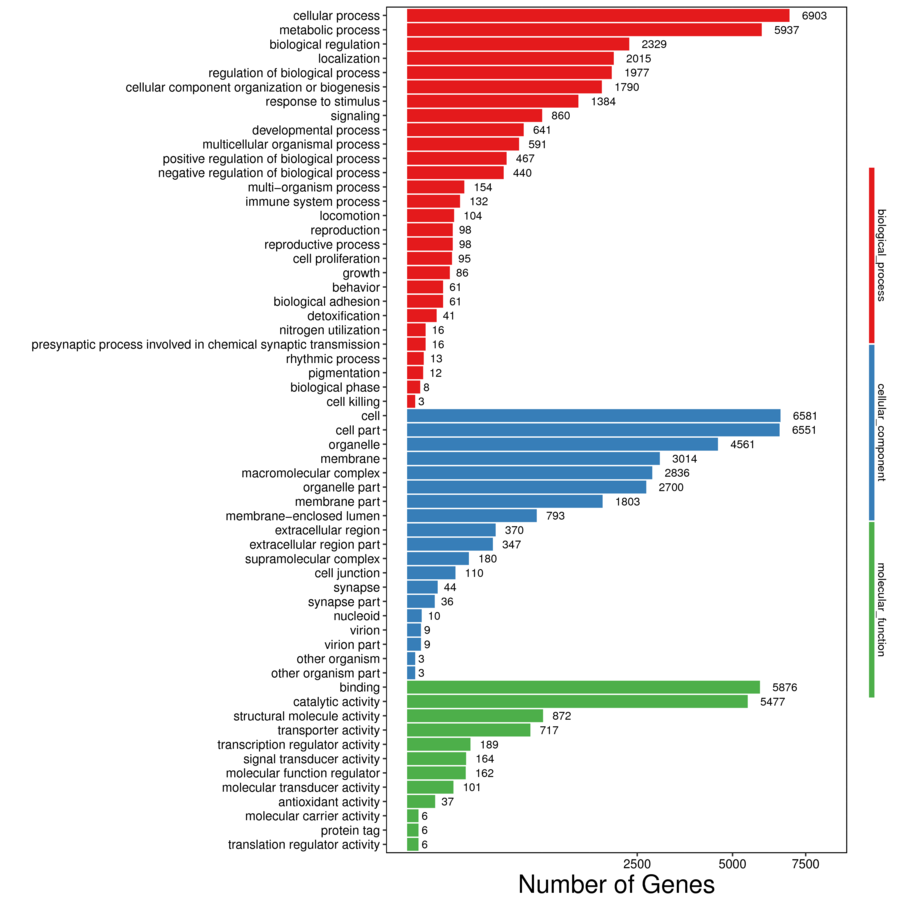


**File 5C**, Gene ontology annotations


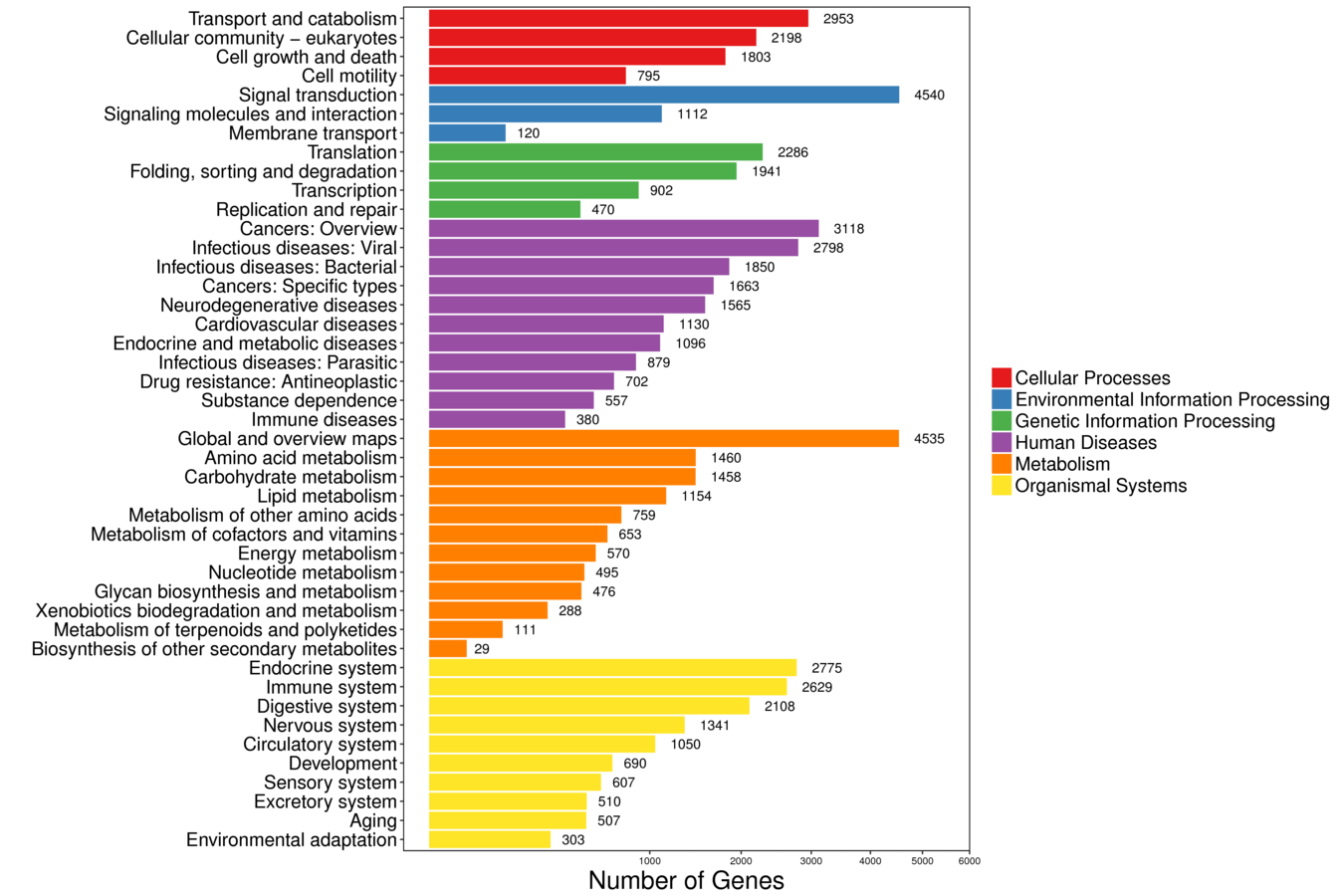


**File 5D**, Functional annotations related to the Kyoto Encyclopedia of Genes and Genomes


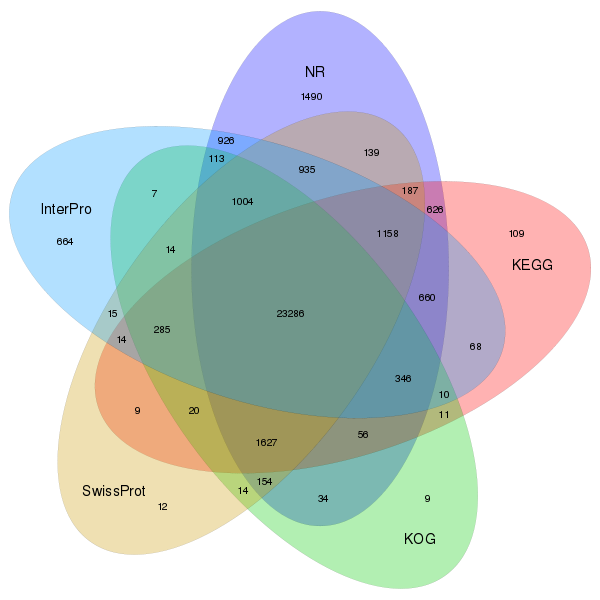


**File 5E**. Venn diagram between NR (non-redundant genes=file 5A), KOG (eukaryotic orthologue groups=file 5B), KEGG(Kyoto Encyclopedia of Genes and Genomes, file 5D), Swissprot and Interpro. 23286 orthogue genes are common to all groups.
