# Supplementary material for: De novo transcriptome assembly of the cubomedusa Tripedalia cystophora, including the analysis of a set of genes involved in peptidergic neurotransmission
Source: BMC Genomics. 2019 Mar 6;20:175. doi: 10.1186/s12864-019-5514-7 (PMC6402141; doi:10.1186/s12864-019-5514-7)
Supplement: Supplementary file 6 — A flow diagram of our software used to predict neuropeptide preprohormones in a transcriptome. (DOCX 137 kb) [file 12864_2019_5514_MOESM6_ESM.docx]

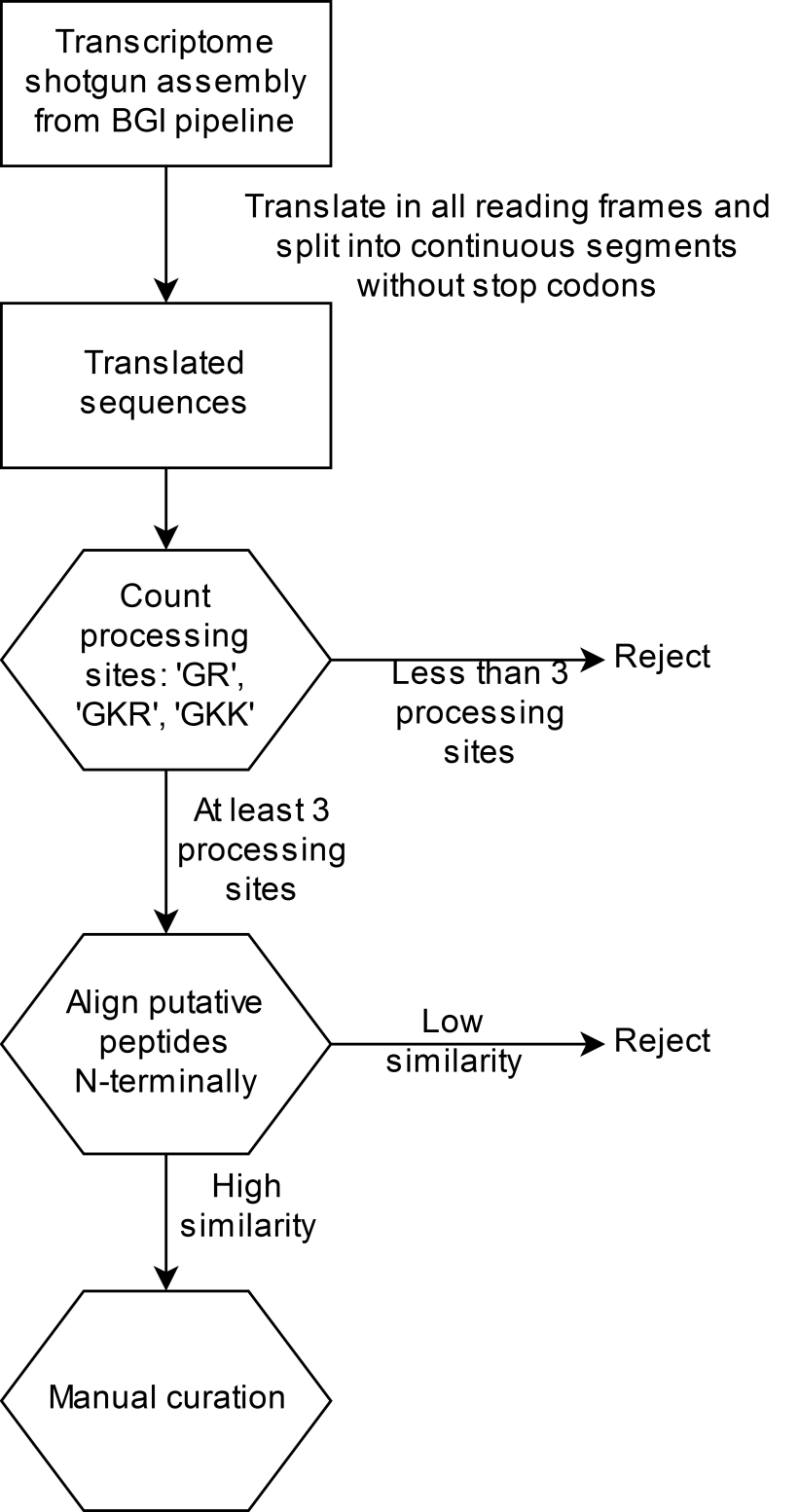


**Additional file 6.** Flowchart of the software program developed to identify neuropeptides in our *T. cystophora* transcriptome. The sequences in the combined transcriptome shotgun assemblies (TSAs) were collected, split into all the possible open reading frames and translated. In each reading frame the number of neuropeptide processing sites (GR, GKR and GKK) was counted. If the count was below the threshold of 3 this particular open reading frame was rejected as a candidate. The putative mature peptides, in the open reading frames with a number of processing sites above the threshold of 3, were aligned. If the peptides had a low level of similarity they were rejected. The program yielded a number of false positives, which were removed by manual curation.
