# Supplementary material for: De novo transcriptome assembly of the cubomedusa Tripedalia cystophora, including the analysis of a set of genes involved in peptidergic neurotransmission
Source: BMC Genomics. 2019 Mar 6;20:175. doi: 10.1186/s12864-019-5514-7 (PMC6402141; doi:10.1186/s12864-019-5514-7)
Supplement: Supplementary file 8 — The amino acid sequences of the predicted RFamideII preprohormones from T. cystophora and A. alata. (DOCX 20 kb) [file 12864_2019_5514_MOESM8_ESM.docx]

**>Tcy-RFamideII**

MKPQTQELLLLITLCISFQNGYSLENSYLQEAFNDANEEFLQCRDMLISSAVKKLVGSRWNDERTVENSDFQKKPRDHFRKRTFNSGNWNQEFPQQFHDIAGELEEIAIQIIAFCRFKILNSNKKDLDAFKRDSYELSHDEAKEKKSILGEGSTESSAKFLKADEPIWSSPAIEVRTKNDRNLRVYTLQELKETNAMIGQHLAAFGILAKKETTGLKPNRRRFGKREVISSASDKIPNRRRFGREVRHGSFSWQYTKKEIPREFVTGENLIRKETPRRRFGKKRDSETDYQRRRFGKRENNENYRKFASLPQNRRFGKREGAENYLRLARIQQNRRFGKRNVDDAKDLGNEYHGKALSDDRGKRDLRSFRRSLFHKDSAFGQAWFKRSKMLAEEDTKSRASNSKRTKAD*

**>Aal-RFamideII**

METLWKVILCCLFLVTCHVGGTDIVQSTTCIGCGNNPLDLEGKHHGITSKPQSISKNPSIMHDKALFEHPRYELEIEKSMRKYGIEKDGFITEFTRQAFQNLEDSVFKYKFYDSDGNTVGNEGSRMESASHPRRYGSSEIAEAPSPLGEVDTSVFLKYLSAFSKKAAEILVAYNELATNNKKVPTAKQIGKRSNVMEKEVPRRFGKRSNEMEEAQPRRFGKRSNEMEEEQLKRFGKRSNEMEDASPRRF…

**Additional file 8.** Amino acid sequences of potential preprohormones from *T. cystophora* and *A. alata*, possibly coding for the dipeptide RFamide. Residues and peptide sequences are highlighted as in Fig. 1. The peptide sequences are preceded by basic residues, which are likely substrates for PC 1/3, resulting in cleavage at the C-terminal sides of R or K and yielding the RFamide dipeptides.
